# Timing dictates efficacy: evaluating the prophylactic and therapeutic potential of dimethyl fumarate against LPS-induced shock

**DOI:** 10.3389/fphar.2026.1966178

**Published:** 2026-10-01

**Authors:** Ahmed Mohammed Mahmood

**Affiliations:** Department of Pharmacology and Toxicology, College of Pharmacy, Al-Mustafa University, Baghdad, Iraq

**Keywords:** acute kidney injury, dimethyl fumarate, lipopolysaccharides, NF-kappa B, oxidative stress

## Abstract

**Background:**

Acute kidney injury (AKI) is a serious and often fatal condition that is caused by endotoxemia as a result of systemic inflammation and uncontrolled oxidative stress. Although dimethyl fumarate (DMF) has shown to have immunomodulatory and antioxidant activity, its specific prophylactic and therapeutic activity in endotoxemia-induced AKI remains unclear.

**Aim:**

to study DMF’s effect on endotoxemia-induced AKI in a mouse *in vivo* model.

**Methods:**

We divided the mice (Swiss albino) into eight equal groups. The endotoxemia-induced AKI was treated by intraperitoneal injection with LPS (5 mg/kg). Prophylactic cohorts received oral DMF (50 or 100 mg/kg/day) for three consecutive days prior to the challenge. Therapeutic cohorts were given a single DMF dose one hour after the treatment. At six hours, gross renal function (BUN, creatinine), cytokine levels, Nrf2/HO-1 activation, oxidative damage biomarkers, inflammasome/apoptotic pathways and acute tubular necrosis (ATN) histopathology were assessed.

**Results:**

LPS challenge resulted in severe renal dysfunction, the onset of a massive cytokine storm and acute tubular destruction. The pre-treatment of DMF prevented the TLR4/NF-κB inflammatory pathway and the overall renal cytoarchitecture was preserved. On the other hand, therapeutic DMF demonstrated a unique anti-inflammatory “ceiling effect” where both low and high doses inhibited NF-κB activation. Therapeutic DMF was more robust in structural and functional rescue, upregulating the Nrf2/HO-1 defense axis, neutralizing reactive oxygen species, and blocking the final inflammasome priming and pyroptotic actions.

**Conclusion:**

The timing of intervention fundamentally dictates pharmacological efficacy. Prophylaxis offers strong preventative shielding, whereas high-dose therapeutic DMF successfully mitigates ongoing oxidative storms, providing a compelling and highly translatable rationale for clinical repurposing in critical care nephrology.

## 1 Introduction

Acute kidney injury (AKI) is a complex disease driven by microvascular dysfunction, oxidative stress, and cellular apoptosis (Li et al., 2024). In particular, tubular epithelial cell damage in AKI is associated with cell cycle arrest, autophagy, and programmed cell death pathways, resulting in the loss of the highly ordered structural integrity of the nephron (Baker and Cantley, 2025).

One of the most powerful and devastating drivers of this endotoxic injury is cytokine release syndrome (CRS), a chronic inflammation of the body that is typically induced by circulating pathogen-associated molecular patterns. Among these is lipopolysaccharide (LPS), a very toxic endotoxin and a significant component of the outer membrane of Gram-negative bacteria (Nozaki et al., 2017). During the LPS-induced CRS, the innate immune system is highly activated and highly reactive. Pathogen-associated molecular patterns, specifically circulating LPS, are directly recognized by Toll-like receptor 4 (TLR4) expressed on the surface of host innate immune cells (i.e., macrophages, monocytes, and dendritic cells in the renal interstitium and systemic circulation). Upon LPS binding, TLR4 activates a rich and complex intracellular signaling cascade involving both MyD88-dependent and TRIF-dependent pathways and key activation factors, such as the nuclear factor of kappa light chain enhancer of B Cells (NF-κB) (Xu et al., 2026; Lou et al., 2025). This activation generates a very fast and massive activation and release of pro-inflammatory cytokines, and thus starts a ‘cytokine storm’. The primary mediators in this initial wave are tumor necrosis factor-alpha (TNF-α) and interleukin-6 (IL-6). In standard rodent models, systemic LPS delivery can induce a quick and profound increase in circulating pro-inflammatory cytokines (Al-Naimi et al., 2025). While exact response time is dependent on dosage, route of administration and species, study results indicate that TNF-α and IL-6 release peaks within 1–3 h of endotoxin exposure for rats (Li et al., 2023; Biesmans et al., 2016; Steven et al., 2017). In addition, in a hyper-inflammatory environment, interleukin-1 beta (IL-1β) and interleukin-18 (IL-18) mediators are also present and are particularly sensitive to early renal tubular damage (Zhou et al., 2024; Yang et al., 2025). In murine models of endotoxemia, targeted deletion of IL-18 receptor signaling has been shown to significantly ameliorate acute tubular necrosis and improve survival; therefore, the immune response acts as a critical driver of structural organ destruction during acute inflammatory states (Nozaki et al., 2017). So, the proper pharmacological management of this cytokine cascade is crucial for preventing and treating LPS-induced AKI.

To prevent the devastating inflammatory cascade in endotoxemia-associated AKI, new drugs for the treatment of immune hyperactivation and preservation of renal tissue are needed. Dimethyl fumarate (DMF), an oral, small-molecule immunomodulatory compound, has been found to be a promising therapeutic candidate (Zhou et al., 2021). It has been approved for relapsing-remitting multiple sclerosis and is used in Europe for treating plaque psoriasis (Al-Jaderi and Maghazachi, 2016; Balak, 2015). DMF has anti-inflammatory and antioxidant properties that are well documented (Yang et al., 2021; Hui et al., 2021).

DMF’s cytoprotective, anti-inflammatory activity has been primarily mediated by two pathways. Firstly, DMF and its metabolite MMF are known to be both electrophilic and antioxidant targets of the nuclear factor erythroid 2-related factor 2 (Nrf2) antioxidant mechanism. In the low-stress setting the Nrf2 is expressed and active but dormant, and the Kelch-like ECH-associated protein 1 (KEAP1) is present in the nucleus and is the molecule that promotes ubiquitination of Nrf2, and thus proteasomal degradation of it is present in the body until it has become ubiquitously and proteasomally degraded (Gao et al., 2024). In addition, DMF has the ability to inhibit pro-inflammatory NF-κB signaling, and its ability to inhibit the pro-inflammatory NF-κB pathway is also very important to the immune system. DMF has been shown to suppress the activation and subsequent nuclear translocation of the NF-κB p65 subunit of the NF-κB p65 target protein and thus inhibit the transcription of pro-inflammatory cytokines, chemokines, and adhesion molecules (Fang et al., 2023). This dual-action mechanism (boosting cellular antioxidant defenses through Nrf2 and silencing inflammatory feed-forward loops via NF-κB) has been explained as a mechanistic basis for its application to autoimmune and inflammatory disorders s (Zingkou et al., 2026; Shihab et al., 2026).

Although the Nrf2 and NF-κB pathways are well-established, the precise therapeutic limits and molecular mechanisms of DMF in the highly specific, hyperacute environment of LPS-induced endotoxemia are presently not well understood. DMF has been shown to prevent tissue fibrosis and improve diabetic nephropathy by reducing systemic inflammation and improving redox balance (Yang et al., 2021). In acute settings, such as ischemia-reperfusion (I/R) injury and cisplatin-induced nephrotoxicity, DMF pretreatment has been shown to significantly decrease elevated levels of blood urea nitrogen (BUN) and serum creatinine (SCr), suppress the upregulation of pro-apoptotic proteins such as Bax and cleaved caspase-3, and visibly improve gross renal histological architecture (Thomas et al., 2022).

Endotoxemia-associated AKI due to an instantaneous cytokine release syndrome can pose a totally different pathological challenge than toxic or ischemic AKI. Macrophage dysfunction and extreme oxidative stress in the renal interstitium are key to this specific pathology (Zhou et al., 2021). Recent transcriptomic studies suggest that the anti-inflammatory effects of DMF are relevant for many immune cell types even in the absence of Nrf2 knockout, and that there are other major upstream regulatory targets for DMF that are unknown (Izumi and Koyama, 2024). The present study aims to understand the effective dose and to explore additional mechanisms for DMF’s cytoprotective effect in septic AKI other than its antioxidant activity from existing antioxidant targets. We believe that the cytoprotective activity of DMF in the treatment of LPS-induced endotoxemia depends on the timing of administration. We assume that prophylactic pre-loading will prevent inflammatory priming in renal cytoarchitecture, and post-insult therapeutic intervention will lead to a different pharmacological response, where antioxidant action will be predominantly based on high-dose anti-inflammatory action to treat an already established cytokine storm. To test this hypothesis in the clinical setting, we compare the molecular, functional, and histopathological outcomes of prophylactic versus therapeutic DMF to understand how the timing of the intervention affects structural rescue and to help develop a model for clinical application in acute critical care nephrology.

## 2 Methods

### 2.1 Chemicals and reagents

Dimethyl fumarate (DMF), thiopental, and dexamethasone (Dexa) were purchased from Sigma-Aldrich, USA. Lipopolysaccharide (LPS) was obtained from Sigma-Aldrich, Germany (*Escherichia coli*, serotype 055: B5, lot 0000133605/99%). Hydroxypropyl methylcellulose (HPMC) was used to prepare the drug vehicle. All other reagents and chemicals were of analytical grade.

### 2.2 Drug formulation and preparation

Because of DMF’s high lipophilicity, it was prepared as a homogeneous suspension for oral administration to ensure accuracy and uniformity. DMF suspension was prepared by dissolving 0.8% (w/v) HPMC in heated distilled water (∼80 °C) with strong magnetic stirring and cooling to room temperature to ensure hydration of the polymer (Traub et al., 2019). DMF powder was prepared before administration. The required amount of DMF powder was placed in a glass mortar, wetted with a few drops of the 0.8% HPMC vehicle, and then triturated to form a smooth paste. The remaining vehicle was added via geometric dilution to achieve the final target concentrations for low-dose and high-dose groups. The final suspensions were continuously agitated on a magnetic stirrer during the whole dosing process. Dexamethasone was also prepared in the 0.8% HPMC vehicle.

### 2.3 Animals, ethical approval, and mitigation of suffering

Adult male Swiss albino mice (n = 64, 25–30 g, 6–8 weeks) were obtained from the Ministry of Health and Environment, Center for Drug Control and Research. The animals were kept inside standard polypropylene cages under conditions of controlled laboratory temperature (22 °C ± 2 °C, humidity 50% ± 10%, and a 12-h light/dark cycle) and were fed and conditioned for 1 week before the experiments. The mice were fed standard laboratory chow and water *ad libitum*. All procedures and handling of the animals were reviewed and approved by the Institutional Animal Care and Use Committee (IACUC) at Al-Mustafa University (Approval No. ALM26-001, 5-2-2026), which is consistent with AVMA 2020 guidelines for the care and use of laboratory animals. The authors follow ARRIVE 2.0 guidelines (Percie du Sert et al., 2020).

We have provided enrichment with environmental materials (nesting materials) to decrease baseline stress. Based on the principle of Refinement, several measures were taken to minimize stress. To control handling-induced stress, all vehicle and therapeutic groups were sham-gavaged during prophylactic loading to prepare the animals to carry out the procedure. All oral (p.o.) gavages and intraperitoneal (i.p.) injections were performed by highly trained personnel using appropriate restraint techniques to minimize duration and pain. After the severe LPS (5 mg/kg) challenge, we kept a visual note of animals during the 6-h acute phase. There were humane endpoints: any animal exhibiting irreversible moribundity (total unresponsiveness to tactile stimuli, excessive respiratory distress (e.g., agonal breathing), or failing to hold up in an upright position prior to the 6-h endpoint) was immediately euthanized using exsanguination with deep thiopental sodium anesthesia.

### 2.4 Experimental design and grouping

To comprehensively evaluate both the mechanism-driven prophylactic efficacy and the clinically relevant therapeutic rescue potential of Dimethyl fumarate (DMF) during an acute cytokine storm, 64 adult male Swiss albino mice were randomly allocated into eight equal cohorts (n = 8). Group 1 was the Normal Control and received oral vehicle and an intraperitoneal (i.p.) normal saline injection. In order to model systemic hyperinflammation, Groups 2 to 8 were challenged with a single i. p. Injection of LPS (5 mg/kg). Group 2 was the untreated Disease Control and received oral vehicle and LPS (Fang et al., 2023; Al-Naimi et al., 2024).

In order to study the prophylactic effect of the treatment, Groups 3, 4, and 5 received oral pre-treatment 3 days before the LPS challenge, twice daily (BID), with Dexamethasone (5 mg/kg/day) (Son et al., 2022), low-dose DMF (50 mg/kg/day), and high-dose DMF (100 mg/kg/day) (Fang et al., 2023; Li et al., 2022), respectively. In addition, to maintain therapeutic levels of drugs during the acute insult, these groups also received a maintenance dose on the day of the challenge (Day 0).

On the contrary, Groups 6, 7, and 8 were first administered the LPS challenge and treated with a single oral dose of Dexamethasone (5 mg/kg) (Son et al., 2022), low-dose DMF (50 mg/kg), or high-dose DMF (100 mg/kg) (Fang et al., 2023; Li et al., 2022), respectively, administered precisely one hour post-insult to intervene during the active onset of the cytokine storm.

### 2.5 Treatment protocol and LPS challenge

All oral treatments were administered by gavage at a volume of 10 mL/kg body weight. To control for handling stress, all mice were handled and gavaged in the same way.

Prophylactic loading phase (days −3 to −1): Groups 3, 4, and 5 were given the drug treatments BID for 3 days prior to the LPS challenge. For this period, to ensure the same handling stress for all animals, groups 1, 2, 6, 7, and 8 were sham-gavaged with the same volume of the 0.8% HPMC vehicle BID.

Challenge and maintenance phase (Day 0): At Time = 0, systemic acute inflammation was induced in groups 2 to 8 by a single i. p. Injection of LPS (5 mg/kg). Group 1 received the same amount of i. p. saline. At T = +1 h post-LPS challenge, the therapeutic groups (Groups 6, 7, and 8) received their active oral drug treatment. At the same time, the prophylactic groups (Groups 3, 4, and 5) had the last continuous dose to ensure active drug coverage during the peak cytokine storm and Groups 1 and 2 received the vehicle (Figure 1).

**FIGURE 1:**
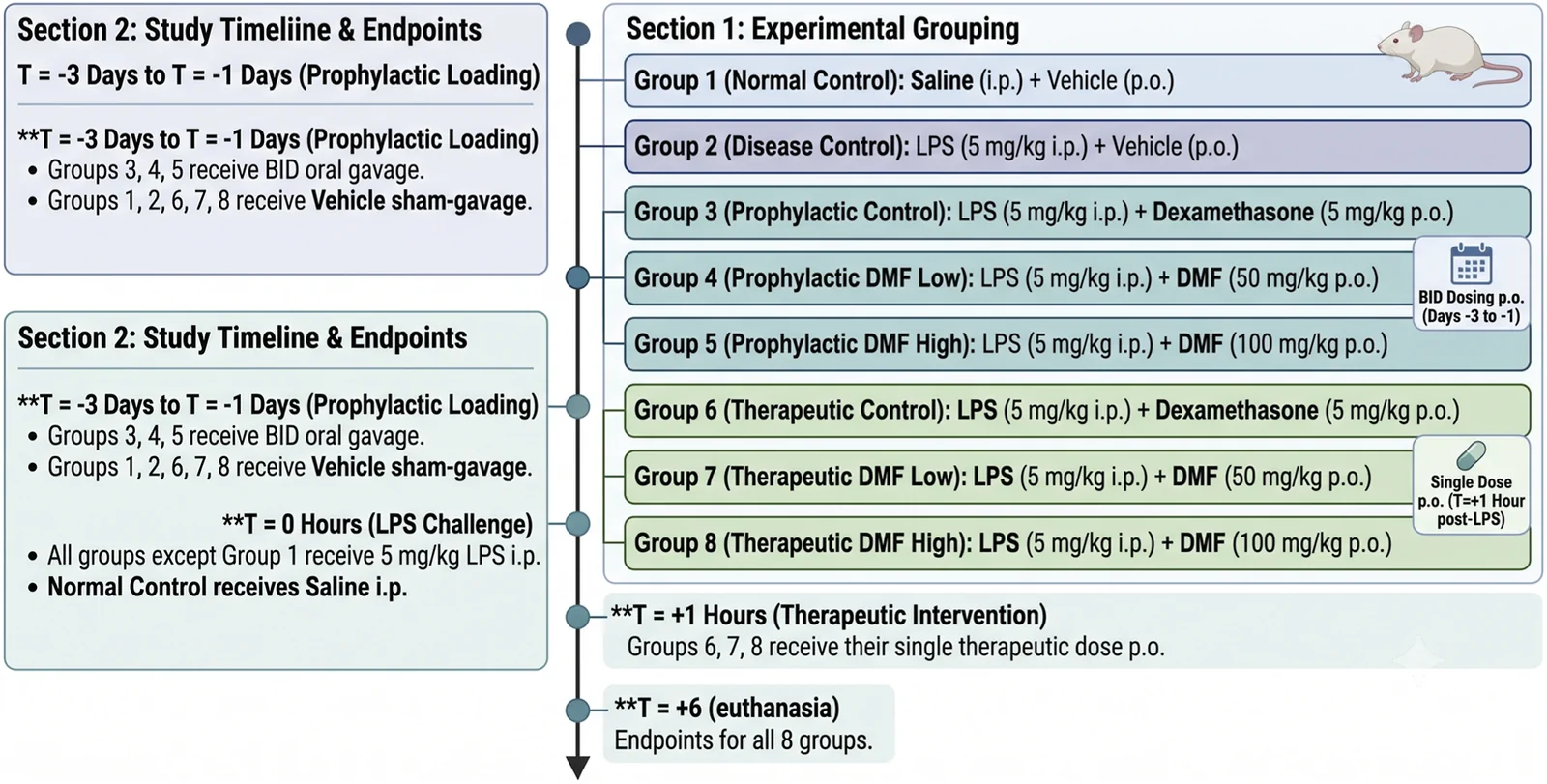
Flowchart and experimental design of the study.

### 2.6 Translation of murine dosages to human equivalent doses (HED)

To assess the clinical relevance and translational viability of our chosen DMF dosages, we scaled the murine levels to Human Equivalent Doses (HED) (Nair and Jacob, 2016). This was done using the Body Surface Area (BSA) normalization approach recommended by the US Food and Drug Administration, using typical allometric scaling factors (Km) for mice (Km = 3) and adult humans (Km = 37) (Nair and Jacob, 2016).

High-Dose Translation (100 mg/kg/day) for a normal 60 kg human corresponds to a dose of ∼486 mg/day, which is consistent with the clinically approved maximum therapeutic dose of oral DMF (Tecfidera) for humans, 480 mg/day (240 mg twice daily), and Low-Dose Translation (50 mg/kg/day) for a normal 60 kg human corresponds to a dose of ∼243 mg/day.

### 2.7 Sample collection and processing

At T = +6 h after the injection of LPS (at the peak of the cytokine storm), all mice were deeply anesthetized with i. p. Injection of thiopental sodium (40 mg/kg) (Alresheedi et al., 2025). Once the surgical plane of anesthesia was established, cardiac puncture was performed to take whole blood, which resulted in terminal exsanguination. Blood was allowed to clot at room temperature for 30 min, and then centrifuged at 3000 × g for 15 min at 4 °C. The separated serum was then carefully aliquoted and stored at −80 °C for enzyme-linked immunosorbent assay (ELISA) quantification of pro-inflammatory cytokines. The kidneys were quickly excised and washed in ice-cold phosphate-buffered saline (PBS). One kidney from each mouse was fixed in 10% neutral buffered formalin for histopathology, while the other was flash-frozen in liquid nitrogen and stored at −80 °C for downstream biochemical and molecular extraction.

### 2.8 Subcellular fractionation and protein extraction

In order to measure the nuclear state of NF-κB (Phospho-p65), flash-frozen kidney tissues were subcellular fractionated with NE-PER™ Nuclear and Cytoplasmic Extraction Reagents (Catalog No. 78833, Thermo Scientific, USA). Briefly, approximately 20 mg of flash-frozen kidney tissue from each subject was washed in cold PBS and homogenized using a Dounce tissue grinder in ice-cold Cytoplasmic Extraction Reagent I (CER I) with Halt™ Protease and Phosphatase Inhibitor Cocktail (Catalog No. 78440, Thermo Scientific, USA) in order to prevent protein degradation and dephosphorylation. The homogenate was then placed on ice for 10 min. After adding Cytoplasmic Extraction Reagent II (CER II), the samples were vortexed and centrifuged at 16,000 × g for 5 min at 4 °C. The finished supernatant (cytoplasmic fraction) was collected. The remaining intact nuclear pellet was added to ice-cold Nuclear Extraction Reagent (NER) supplemented with the protease/phosphatase inhibitor cocktail, vortexed multiple times over 40 min on ice, and centrifuged at 16,000 × g for 10 min at 4 °C. The highly enriched nuclear protein fraction was immediately frozen and stored at −80 °C. To measure total tissue biomarkers, a fraction of flash-frozen kidney tissue was homogenized in standard ice-cold RIPA lysis buffer and then centrifuged at 12,000 × g for 15 min at 4 °C to obtain the total protein supernatant. Total protein concentrations for all nuclear and total tissue fractions were determined using a Pierce™ BCA Protein Assay Kit (Catalog No. 23225, Thermo Scientific, USA) to ensure equal protein mass loading and normalization during subsequent ELISA and colorimetric analyses (Fan et al., 2020; Ye et al., 2015).

### 2.9 RNA extraction and quantitative real-time PCR (RT-qPCR)

Total RNA was extracted from flash-frozen kidney tissue using the TransZol Up Plus RNA Kit (Catalog No. ET111-01, TransGen Biotech, China) according to the manufacturer’s instructions. RNA concentration and purity were assessed spectrophotometrically by measuring absorbance at 260 and 280 nm, and samples with an A260/A280 ratio between 1.8 and 2.0 were considered acceptable for further analysis. First-strand complementary DNA (cDNA) was synthesized using the EasyScript® First-Strand cDNA Synthesis SuperMix (Catalog No. AE311-02, TransGen Biotech, China). For each sample, a 20 µL reaction mixture containing 1 µL random primer (N9), 10 µL 2× ES reaction mix, 1 µL EasyScript® RT/RI enzyme mix, 3 µL RNase-free water, and 5 µL total RNA was prepared. Reverse transcription was performed at 25 °C for 11 min, followed by 42 °C for 35 min, and enzyme inactivation at 85 °C for 5 min. The synthesized cDNA was stored at −20 °C until quantitative PCR analysis. All reactions were performed in duplicate (Bustin et al., 2009).

Quantitative real-time PCR was carried out using GoTaq® qPCR Master Mix (Catalog No. A6001, Promega, USA) on a SaCycler-96 Real-Time PCR System (Sacace, Italy). Each PCR reaction was prepared in a final volume of 25 µL containing 10 µL of 2× GoTaq® qPCR Master Mix, 1 µL of forward primer (10 pmol/μL), 1 µL of reverse primer (10 pmol/μL), 2 µL of cDNA template, and 11 µL of nuclease-free water. The gene-specific primers used in this study are illustrated in Table 1. Glyceraldehyde-3-phosphate dehydrogenase (Gapdh) was utilized as the stable internal reference gene based on its stable expression across experimental groups. Primer sequences were designed based on murine (*Mus musculus*) reference mRNA sequences obtained from the NCBI GenBank database and validated *in silico* using NCBI BLAST to confirm gene-specific amplification. Primers were designed to span exon–exon junctions whenever possible to minimize amplification of genomic DNA (Bustin et al., 2009; Kassid et al., 2026).

**TABLE 1:** Murine (*Mus musculus*) RT-qPCR primer sequences and GenBank accession numbers.

Gene symbol	GenBank accession	Forward primer (5'→ 3′)	Reverse primer (5'→ 3′)
Gapdh	NM_008084	AGG​TCG​GTG​TGA​ACG​GAT​TTG	GGG​GTC​GTT​GAT​GGC​AAC​A
Tlr4	NM_021297	ATG​GCA​TGG​CTT​ACA​CCA​CC	GAG​GCC​AAT​TTT​GTC​TCC​ACA
Hmox1 (HO-1)	NM_010442	AAG​CCG​AGA​ATG​CTG​AGT​TCA	GCC​GTG​TAG​ATA​TGG​TAC​AAG​GA
Nqo1	NM_008706	AGG​ATG​GGA​GGT​ACT​CGA​ATC	TGC​TAG​AGA​TGA​CTC​GGA​AGG
Bax	NM_007527	TGA​AGA​CAG​GGG​CCT​TTT​TG	AAT​TCG​CCG​GAG​ACA​CTC​G
Bcl2	NM_009741	GTC​GCT​ACC​GTC​GTG​ACT​TC	CAG​ACA​TGC​ACC​TAC​CCA​GC
Txnip	NM_023719	TCG​GTG​TGT​GAA​GTT​AAC​CCC	CGA​AGG​ACC​TCC​TGT​TTG​AGC
Nlrp3	NM_145,827	ATT​ACC​CGC​CCG​AGA​AAG​G	TCG​CAG​CAA​AGA​TCC​ACA​CAG
Tnf	NM_013693	TTA​AGT​GTG​TGT​CCC​TGA​GGT	CCA​AGA​GCA​GCC​CAA​CAA​ATC
Il6	NM_031168	GCC​ACT​GCC​TTC​CCT​ACT​TC	TCT​GAC​AGT​GCA​TCA​TCG​CT
Il1b	NM_008361	GCA​ACT​GTT​CCT​GAA​CTC​AAC​T	ATC​TTT​TGG​GGT​CCG​TCA​ACT

PCR amplification was performed with an initial denaturation step at 95 °C for 2 min, followed by 40 cycles consisting of denaturation at 95 °C for 15 s and annealing/extension at 60 °C for 30 s. A melt-curve analysis was conducted immediately after amplification by gradually increasing the temperature from 65 °C to 95 °C, with increments of 0.5 °C every 5 s, to confirm amplification specificity. The presence of a single sharp melt-curve peak indicated specific amplification of the target genes. No-template controls (NTC) were included in each run to exclude reagent contamination, and no-reverse transcription controls (NRT) were included to exclude genomic DNA contamination. Relative gene expression levels were calculated using the 2^−ΔΔCt^ method. The ΔCt value was determined by subtracting the Ct value of the reference gene (Gapdh) from that of the target gene, while ΔΔCt was calculated by subtracting the mean ΔCt of the control group from the ΔCt of each experimental sample (Livak and Schmittgen, 2001).

### 2.10 ELISA and biochemical assays

The stored serum samples, total kidney tissue homogenates (RIPA), and highly enriched nuclear fractions (NE-PER) were thawed and quantified by targeted biomolecular measurements. All analyses were done according to the manufacturers’ instruction manuals, and the samples and standards were run in duplicate. The OD readings were measured on a microplate reader.

#### 2.10.1 Standard sandwich ELISAs

To quantify the levels of specific cytokines, signaling proteins, and structural injury markers, standard sandwich ELISA kits are widely used. These included the Quantikine® ELISA kits by R&D Systems, USA (for Serum TNF-α [Catalog No. MTA00B], IL-6 [Catalog No. M6000B], IL-1β [Catalog No. MLB00C], and tissue KIM-1 [Catalog No. MKM100]); PathScan® ELISA kits from Cell Signaling Technology, USA (for tissue Nuclear Phospho-Ser536 p65 [Catalog No. 7173], Cleaved Gasdermin D (Asp276) (Catalog No. #25566), and tissue Cleaved Caspase-3 [Catalog No. 7190]); and SimpleStep ELISA® from Abcam, United Kingdom (for tissue HO-1 [Catalog No. ab204524]). Although they are focused on different proteins, these assays share the same sandwich immunoassay principle. Standards and prepared samples (diluted as required for assay optimization) were added to 96-well microplates pre-coated with highly specific capture antibodies and incubated for 1–2 h at room temperature. After washing using the buffer provided to remove any unbound material, biotinylated or HRP-conjugated detection antibodies were added to each well and incubated. Streptavidin-Horseradish Peroxidase (if applicable) and a TMB (3,3′,5,5′-tetramethylbenzidine) substrate solution were added at a subsequent time in order to induce a colorimetric reaction with the target protein concentration. The reaction was terminated using an acidic stop solution, and absorbance was measured at 450 nm with a reference wavelength at 540 nm or 570 nm, as specified by the manufacturer. The target concentrations are interpolated from a four-parameter logistic (4PL) standard curve (Mahmood et al., 2024).

#### 2.10.2 Nrf2 transcription factor DNA-Binding activity assay

To confirm the translocation and DNA-binding activity of Nrf2, we used the TransAM® Nrf2 Kit (Catalog No. 50296, Active Motif, USA) on the extracted nuclear fractions. Unlike sandwich ELISAs that measure the total amount of protein present in a sample, this assay measures functional activity. The 96-well plate was coated with an oligonucleotide with the Antioxidant Response Element (ARE) consensus binding sequence (5′-GTC​ACA​GTG​ACT​CAG​CAG​AAT​CTG-3′). The extracted nuclear fractions were poured into the drilled wells and incubated for 24 h to allow Nrf2 to physically bind to the immobilized DNA sequence. After washing to remove inactive/unbound proteins, a primary antibody recognizing an accessible epitope on the bound Nrf2 protein was added. The secondary antibody was then conjugated to HRP and developed colorimetrically with TMB substrate and stopped with stop solution. Absorbance was read at 450 nm, providing a direct reference for Nrf2 activation.

#### 2.10.3 Competitive enzyme immunoassay for oxidative DNA damage (8-OHdG)

8-hydroxy-2′-deoxyguanosine (8-OHdG) levels, a vital biomarker for oxidative DNA damage, were measured in kidney tissue lysates using the DNA/RNA Oxidative Damage ELISA kit (Catalog No. 589320, Cayman Chemical, USA). The assay is based on competitive binding. Briefly, the tissue samples were incubated with an 8-OHdG-acetylcholinesterase (AChE) conjugate (the Tracer) and an 8-OHdG-specific monoclonal antibody. The 8-OHdG present in the tissue sample competes with the Tracer for only a few antibody binding sites. After washing the plate to remove unbound reagents, Ellman’s Reagent (which contains the substrate for AChE) was added. The enzymatic reaction produced a yellow color, which was measured at 412 nm. Because of the competitive nature of the assay, the intensity of the color was inversely proportional to the concentration of free 8-OHdG in the sample.

#### 2.10.4 Colorimetric biochemical assays

To assess gross functional renal impairment, tissue antioxidant capacity, lipid peroxidation, and pyroptosis execution, specific colorimetric assay kits were used. BUN and Creatinine were measured using the BUN Colorimetric Detection Kit (Catalog No. EIABUN, Invitrogen, USA) and the Creatinine Colorimetric Assay Kit (Catalog No. EIACREA, Invitrogen, USA). Total Superoxide Dismutase (SOD) activity in the total kidney homogenate was measured with the SOD Assay Kit (Catalog No. 706002, Cayman Chemical, USA). We measured lipid peroxidation by measuring MDA levels with the Lipid Peroxidation (MDA) Assay Kit (Catalog No. ab118970, Abcam, United Kingdom).

We also determined the enzymatic activity of cleaved Caspase-1 in tissue lysates using the Caspase-1 Colorimetric Assay Kit (Catalog No. ab39411, Abcam, United Kingdom). Shortly, these assays are based on proportional biochemical reactions that produce a visible color change to be read by spectrophotometry. For renal function, we used the kit setup to react with specific substrates to form chromogenic complexes according to the metabolite concentration. In order to measure SOD activity, we used tissue lysates with tetrazolium salt and xanthine oxidase; the SOD in the tissue does not allow the tetrazolium salt to be reduced by superoxide radicals. For MDA, we used tissue homogenates boiled down to 95 °C for 60 min to make an MDA-TBA adduct and then incubated them with a synthetic peptide substrate of Caspase-1, which is engineered to release the chromophore p-nitroanilide (pNA). After the ideal incubation times specified by the manufacturers, absorbance was measured with a microplate reader at the appropriate wavelengths (405 nm for Caspase-1, 450 nm for SOD, 490 nm for creatinine, and 532 nm for MDA-TBA adduct). Analyte concentrations or enzymatic activity were then compared with standard curves generated on the same plate or fold changes relative to control samples.

### 2.11 Histopathology and semi-quantitative renal injury scoring

#### 2.11.1 Tissue processing and H&E staining

To determine structural renal damage, the formalin-fixed kidney tissues (24–48 h in 10% neutral buffered formalin) were dehydrated with a number of ascending ethanol levels (70%, 80%, 90%, and 100%), cleared in xylene, and embedded in paraffin wax blocks. Using a rotary microtome, the paraffin blocks were sectioned at 5 µm thickness. The sections were mounted on glass slides and deparaffinized in xylene and then rehydrated with lower levels of ethanol to distilled water. The slides were then stained with Hematoxylin and Eosin (H&E) to measure the overall structure, nuclear details, and cytoplasmic integrity of the tissue. The slides were dried, cleared, and stored with a synthetic resinous mounting material.

#### 2.11.2 Semi-quantitative scoring of acute tubular necrosis (ATN)

Histopathological evaluation was performed using a light microscope (Olympus, Japan) equipped with a digital imaging system. To eliminate observer bias, the evaluation was conducted by an experienced veterinary pathologist who was entirely blinded to the experimental treatment groups.

The severity of LPS-induced Acute Kidney Injury was assessed using a validated semi-quantitative scoring system focused on the hallmarks of Acute Tubular Necrosis (ATN). Ten randomly selected non-overlapping, high-power fields (HPFs, ×400 magnification) within the renal cortex and outer medulla were evaluated per slide. The damage was graded based on the presence of the following morphological features: tubular epithelial cell vacuolization, tubular dilation, flattening of the tubular epithelium, loss of the brush border, formation of intratubular proteinaceous casts, interstitial inflammatory cell infiltration, and frank cellular necrosis/sloughing.

Each field was assigned a score based on the percentage of the tubular area exhibiting these signs of injury:

- Score 0: Normal histology (0% damage).
- Score 1: Mild injury (Damage involving <25% of the affected field).
- Score 2: Moderate injury (Damage involving 25%–50% of the affected field).
- Score 3: Severe injury (Damage involving 50%–75% of the affected field).
- Score 4: Extensive injury (Damage involving >75% of the affected field).

The scores from the ten fields were averaged for each mouse to generate a single composite ATN score for statistical comparison across the experimental cohorts.

### 2.12 Sample size calculation and randomization

The sample size was determined *a priori* by G*Power software. In order to achieve 80% statistical power (1 - β = 0.80) with significance level α = 0.05 and effect size (Cohen’s f = 0.40) for a multi-group ANOVA, at least six to seven mice were chosen for each group. To control for potential loss of population due to the severe LPS challenge, the sample size was also changed to n = 8 for each group to achieve a total of 64 mice for the study. After 1 week of acclimatization, mice were randomly assigned to groups based on their body weight to ensure that the same weights were received for all groups. The group assignment (Groups 1–8) was generated by a computer using a random number generator. To reduce bias, researchers conducting the biochemical analyses were blinded to the treatment group allocation.

### 2.13 Statistical analysis

All statistical analyses were performed using GraphPad Prism software (Version 11, GraphPad Software, USA). To ensure the appropriateness of parametric testing, the distribution of quantitative datasets was first evaluated for normality using the Shapiro-Wilk test. Following confirmation of a normal distribution, the data were expressed as the mean ± standard deviation (SD). Differences among the experimental cohorts for all biochemical, molecular, and functional endpoints (e.g., BUN, Creatinine, KIM-1, Cytokines, and RT-qPCR data) were evaluated using a one-way analysis of variance (ANOVA).

To specifically identify significant differences between the multiple treatment groups and their respective controls while maintaining stringent control over the family-wise error rate, Holm-Šídák’s post-hoc multiple comparisons test was utilized. The semi-quantitative Acute Tubular Necrosis (ATN) histopathological scores, derived from multiple high-power fields per subject, were similarly analyzed using a one-way ANOVA followed by Holm-Šídák’s post-hoc test. For all analyses, a two-tailed p-value of less than 0.05 ( p<0.05 ) was considered statistically significant.

## 3 Results

### 3.1 Timing-dependent efficacy of dimethyl fumarate on LPS-induced mortality

To address the survival outcomes of endotoxemic shock and avoid survivor bias, a Kaplan-Meier survival analysis and a Cox proportional hazards regression were conducted across all experimental cohorts (N = 90), with mortality as the primary experimental endpoint over the 6-h acute phase. The unmitigated lipopolysaccharide challenge in the Disease Control cohort triggered rapid and severe attrition, acting as the baseline reference for the maximum hazard of death (Table 2; Figure 2).

**TABLE 2:** Cox proportional hazards regression analysis of timing-dependent dimethyl fumarate interventions on LPS-Induced mortality.

Experimental group	Hazard ratio (HR)	95% Confidence interval (CI)	P-value
Disease control (LPS)	1.00 (reference)	—	—
Normal control	Not calculable*	—	0.0005
Prophylactic dexa	0.108	0.006–0.568	0.0052
Prophylactic low DMF	0.203	0.031–0.773	0.0173
Prophylactic high DMF	Not calculable*	—	0.0005
Therapeutic dexa	0.287	0.064–0.940	0.0386
Therapeutic low DMF	0.506	0.172–1.367	0.1803
Therapeutic high DMF	0.361	0.099–1.083	0.0697

* Hazard ratios and 95% CIs, are not calculable for cohorts with 100% survival due to the mathematical absence of mortality events (complete separation). The reported p-values for these specific groups were derived independently using the Kaplan-Meier Log-rank (Mantel-Cox) test.

**FIGURE 2:**
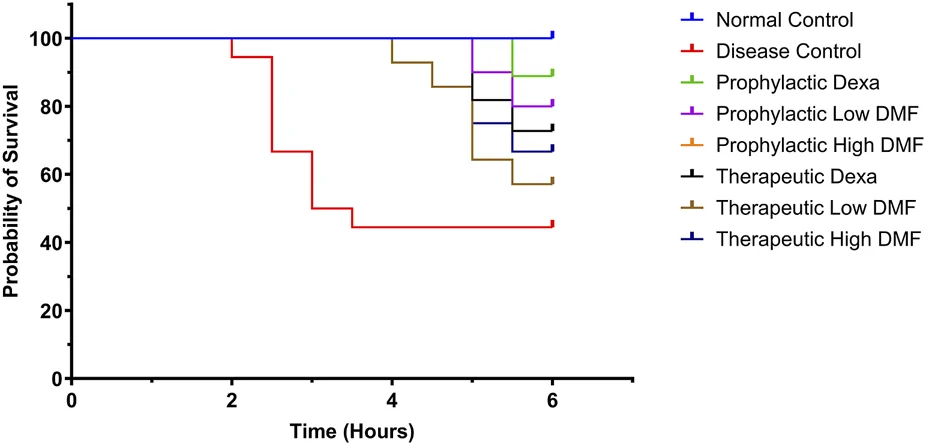
Kaplan-Meier survival analysis demonstrating the timing-dependent protective efficacy of Dimethyl Fumarate (DMF) against LPS-induced endotoxemic shock.

The high-dose prophylactic regimen (100 mg/kg) prevented early death and had 100% survival (p = 0.0005). In addition, the low-dose prophylactic regimen (50 mg/kg) was more effective in reducing risk of death by 80% versus the untreated Disease Control (HR = 0.203; 95% CI: 0.031–0.773; p = 0.0173). This safety benefit was also comparable to the established safety benefits of Dexamethasone Prophylactic Control (HR = 0.108; 95% CI: 0.006–0.568; p = 0.0052).

The Dexamethasone Therapeutic Control reduced the risk of death by 64% (HR = 0.287; 95% CI 0.064–0.940, p = 0.0386). Therapeutic Dimethyl fumarate did not show a significant effect on mortality. The high-dose therapy showed a 64% decrease in mortality hazard (HR = 0.361; 95% CI 0.099–1.083, p = 0.0697) and low-dose therapy did not significantly lower the probability of death compared to untreated patients (HR = 0.506; 95% CI 0.172–1.367, p = 0.1803).

These survival metrics precisely parallel the established biochemical findings. They definitively prove that while post-insult therapeutic Dimethyl fumarate actively clears reactive oxygen species to mitigate localized structural renal damage, preemptive prophylactic loading is fundamentally required to silence the upstream NF-κB inflammatory switch and completely prevent the terminal execution of systemic LPS-induced shock.

### 3.2 DMF attenuates LPS-induced acute renal dysfunction and structural injury

In the presence of endotoxemia in the LPS (Disease Control group), the acute renal impairment was clear from the increase in BUN to 7.05 ± 0.72 mg/dL from 0.74 ± 0.08 mg/dL in Normal Controls (p < 0.0001; Figure 3A). The same is true for serum creatinine (13.72 ± 1.19 mg/dL and 1.42 ± 0.11 mg/dL in Normal Controls, p < 0.0001; Figure 3B). We also quantified tubular injury and found that tissue KIM-1 levels were significantly increased to 1395.0 ± 136.68 pg/mL from the basal level of 118.0 ± 12.0 pg/mL (p < 0.0001; Figure 3C).

**FIGURE 3:**
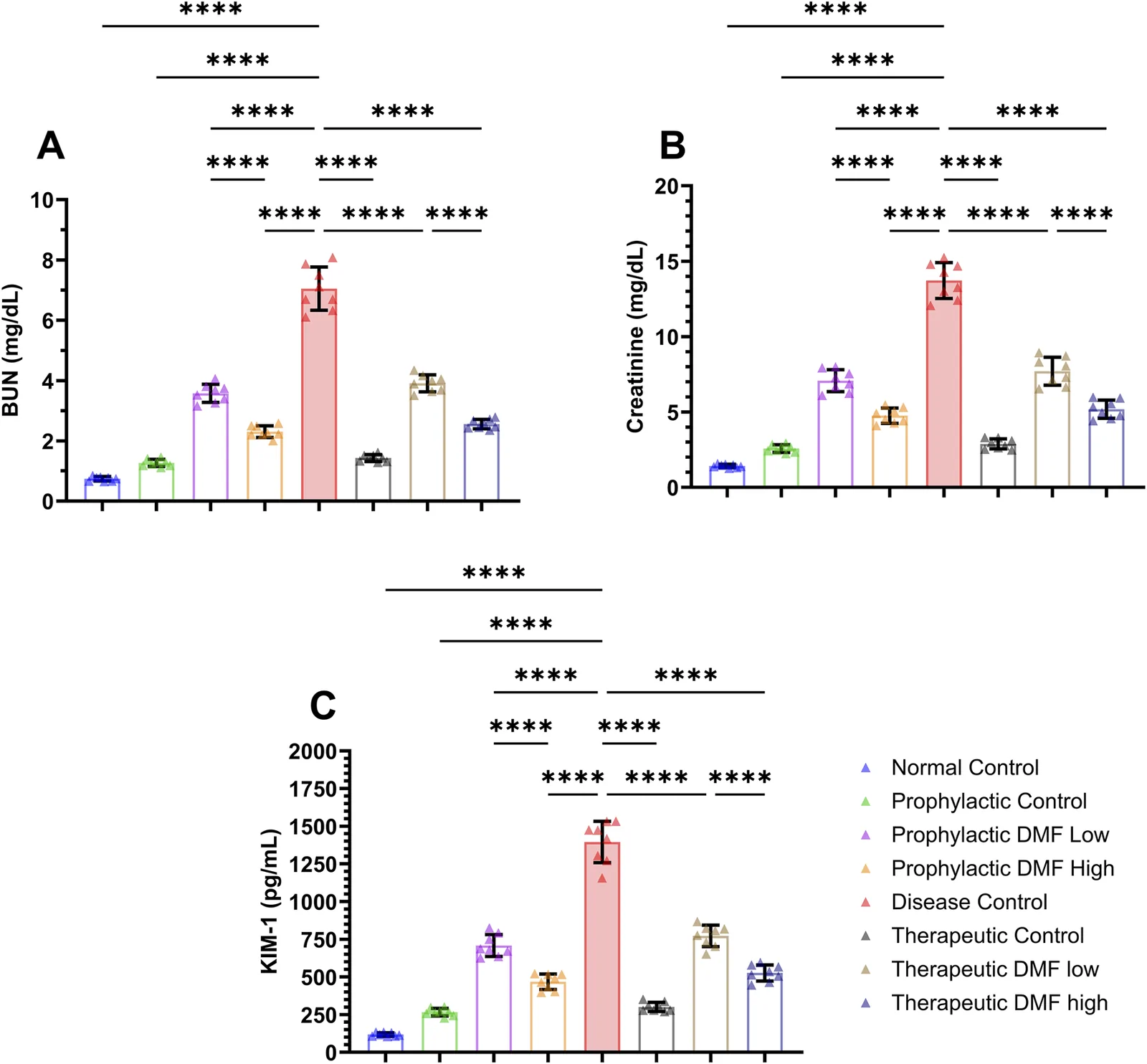
DMF attenuates LPS-induced acute renal dysfunction and structural tubular damage. The effect of prophylactic and therapeutic treatments on systemic and structural injury marker levels: **(A)** BUN (mg/dL), **(B)** serum creatinine (mg/dL), **(C)** tissue KIM-1 (pg/mL). Data are presented as individual replicate values overlaid on mean ± SD (n = 8 subjects per group). Statistical variance was evaluated using a one-way ANOVA followed by Holm-Šídák’s multiple comparisons test (****p < 0.0001) to assess the variance between groups.

Administration of DMF in both prophylactic and therapeutic modalities significantly mitigated LPS-induced nephrotoxicity in a dose-dependent manner. In the prophylactic arm, high-dose DMF significantly attenuated the elevation of renal injury markers, limiting BUN levels to 2.31 ± 0.19 mg/dL (p < 0.0001 vs. Disease Control; Figure 3A), serum creatinine to 4.76 ± 0.50 mg/dL (p < 0.0001; Figure 3B), and tissue KIM-1 levels to 468.0 ± 51.27 pg/mL (p < 0.0001; Figure 3C). The low-dose prophylactic regimen demonstrated a similar, albeit less pronounced, protective effect against LPS-induced elevations (BUN: 3.58 ± 0.30 mg/dL, Figure 3A; serum creatinine: 7.08 ± 0.72 mg/dL, Figure 2B; tissue KIM-1: 708.0 ± 72.94 pg/mL, Figure 3C).

Correspondingly, therapeutic administration initiated post-LPS challenge significantly attenuated ongoing renal deterioration. High-dose therapeutic DMF yielded a statistically significant reduction in BUN (2.56 ± 0.16 mg/dL; Figure 3A), serum creatinine (5.19 ± 0.60 mg/dL; Figure 3B), and tissue KIM-1 (526.0 ± 53.67 pg/mL; Figure 3C) relative to the untreated Disease Control cohort (p < 0.0001). Vehicle-treated prophylactic and therapeutic control groups exhibited baseline equivalence or expected unmitigated injury profiles, verifying that the observed renal preservation is attributable to the specific pharmacological intervention of DMF.

### 3.3 DMF suppresses TLR4/NF-κB-mediated systemic and local inflammation

To investigate the inflammatory mechanisms behind LPS-induced renal injury, we examined the TLR4/NF-κB axis. LPS (Disease Control group) stimulated significant transcriptional activation of the master inflammatory receptor, with Tlr4 mRNA expression up 8.60 ± 1.04-fold higher than the baseline of 1.00 ± 0.11 in Normal Controls (p < 0.0001; Figure 4A). In addition to the transcriptional activation, the main transcription factor was dramatically translocated to nuclear levels (Phospho-p65, 4.52 ± 0.23 compared to 0.39 ± 0.05 in normal controls with a p < 0.0001 value; Figure 4B). This was accompanied by a severe local and systemic inflammatory response. mRNA levels of Tnf, Il6, and Il1b increased in renal tissue to 9.48 ± 1.03, 10.28 ± 1.15, and 9.81 ± 1.17-fold from normal control (p < 0.0001; Figures 4F–H). In addition to this localized renal response, systemic serum levels of TNF-α (490.00 ± 48.21 pg/mL), IL-6 (564.00 ± 59.11 pg/mL), and IL-1β (455.00 ± 63.77 pg/mL) showed significantly elevated values compared with clinical values (p < 0.0001; Figures 4C–E).

**FIGURE 4:**
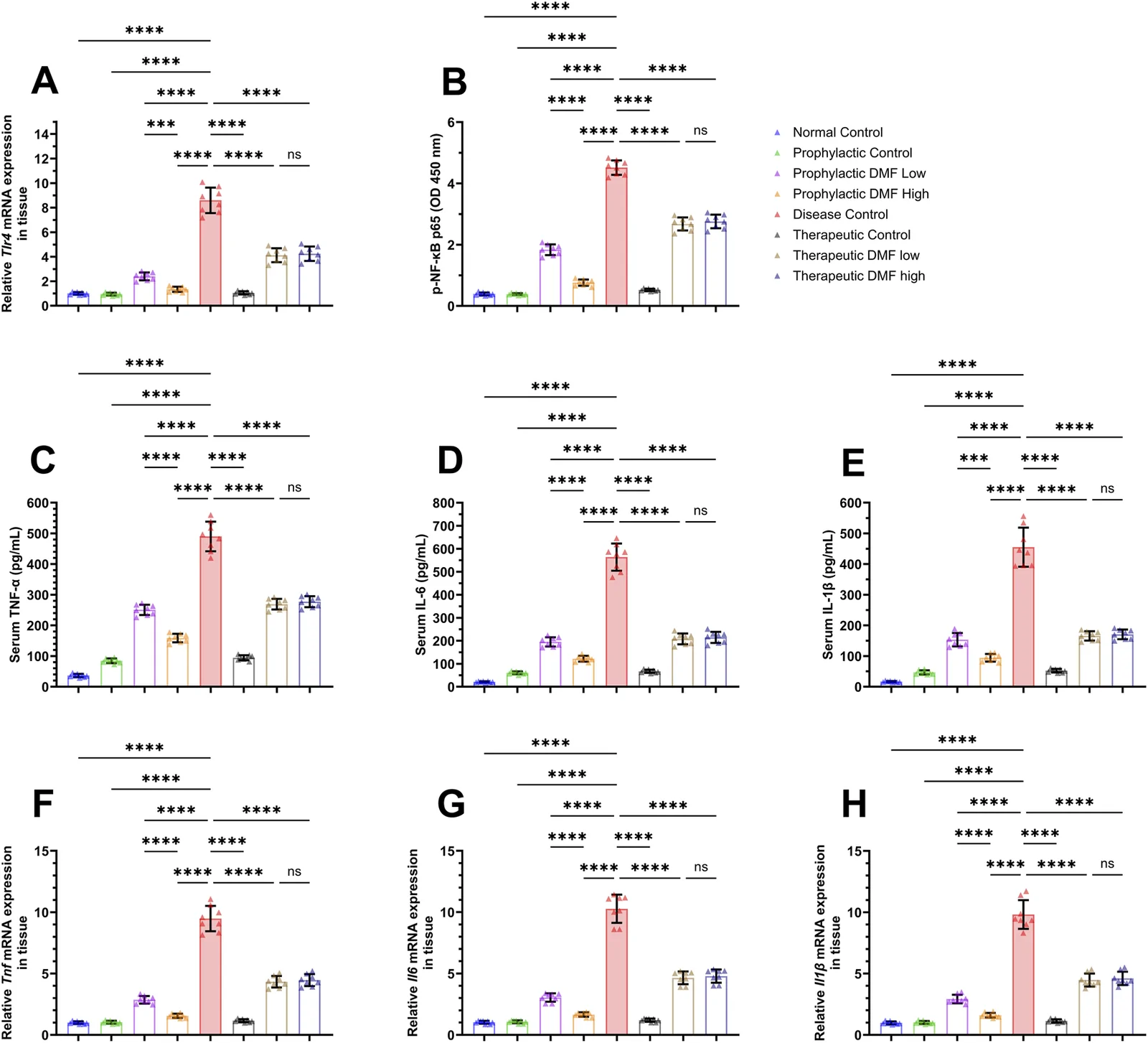
DMF suppresses TLR4/NF-κB-mediated local and systemic inflammation. The effects of prophylactic and therapeutic DMF treatments on the inflammatory cascade. Tissue mRNA expression of **(A)** Tlr4, **(F)** Tnf, **(G)** Il6, **(H)** Il1b are expressed as relative fold change. Protein levels of **(B)** Phospho-p65 (Ser536) were measured by Sandwich ELISA (OD 450 nm). Systemic circulating cytokine levels of **(C)** serum TNF-α, **(D)** serum IL-6, **(E)** serum IL-1β are presented in pg/mL. The representative values of the replicates are plotted as individual replicate values, with the mean ± SD (n = 8 subjects in each group). The statistical variance was computed in a one-way ANOVA followed by Holm-Šídák’s multiple comparisons test (e.g., p < 0.0001 and ***p < 0.001 are significant differences between the groups; ns indicates no significant difference (p > 0.05).

The DMF treatment was very effective in blocking this inflammatory cascade in a dose-dependent manner. The high-dose prophylactic DMF suppressed Tlr4 expression significantly (1.34 ± 0.20-fold in our study) (p < 0.0001) and NF-κB activation (0.76 ± 0.10) (p < 0.0001) by reducing Phospho-p65 to 0.76 ± 0.10 (p < 0.0001) (Figure 4B). This upstream blockade dramatically decreased local cytokine transcription (Figures 4F–H) and caused a reduction of circulating systemic cytokines (Figures 4F–H) and led to TNF-α to 158.71 ± 13.86 pg/mL (p < 0.0001) in serum (Figure 4C).

DMF treatment had a similar effect on the TLR4/NF-κB axis and downstream cytokines compared to the Disease Control group (p < 0.0001). However, unlike the prophylactic approach, the anti-inflammatory action of DMF exhibited a distinct plateau. At low and high doses, the inflammatory markers were comparable across Tlr4 (4.12 ± 0.57 vs. 4.24 ± 0.58, p > 0.05; Figure 4A), Phospho-p65 (2.76 ± 0.22 vs. 2.68 ± 0.21, p > 0.05; Figure 4B), tissue cytokine mRNA (Figures 4F–H), and circulating cytokines (Figures 4C–E). It is evident that the saturation in DMF demonstrates a ceiling effect at the lower therapeutic dose, while the main therapeutic activity at higher doses relies predominantly on antioxidant mechanisms.

### 3.4 Activation of the Nrf2/HO-1 antioxidant defense axis

To delineate the pharmacological mechanism by which the intervention upregulates internal cellular defenses against LPS-induced oxidative stress, the Nrf2/HO-1 antioxidant signaling axis was evaluated. In the Disease Control group, LPS intoxication triggered a compensatory but insufficient endogenous antioxidant response. This was characterized by a moderate increase in Nrf2 DNA-binding activity to an OD of 1.01 ± 0.12 compared to the baseline of 0.39 ± 0.05 in Normal Controls (p < 0.0001; Figure 5A). Consequently, downstream transcription of antioxidant genes was partially elevated, with Hmox1 and Nqo1 mRNA reaching 2.20 ± 0.17-fold and 2.58 ± 0.27-fold, respectively (Figures 5B,C). This translated to an accumulation of HO-1 protein to 613.13 ± 93.52 pg/mL (p < 0.0001; Figure 5D). Despite this upregulation, the severe oxidative burden rapidly exhausted the primary functional cellular defense, causing endogenous SOD activity to plummet from a normal baseline of 0.038 ± 0.003 U/mL down to 0.007 ± 0.001 U/mL (p < 0.0001; Figure 5E).

**FIGURE 5:**
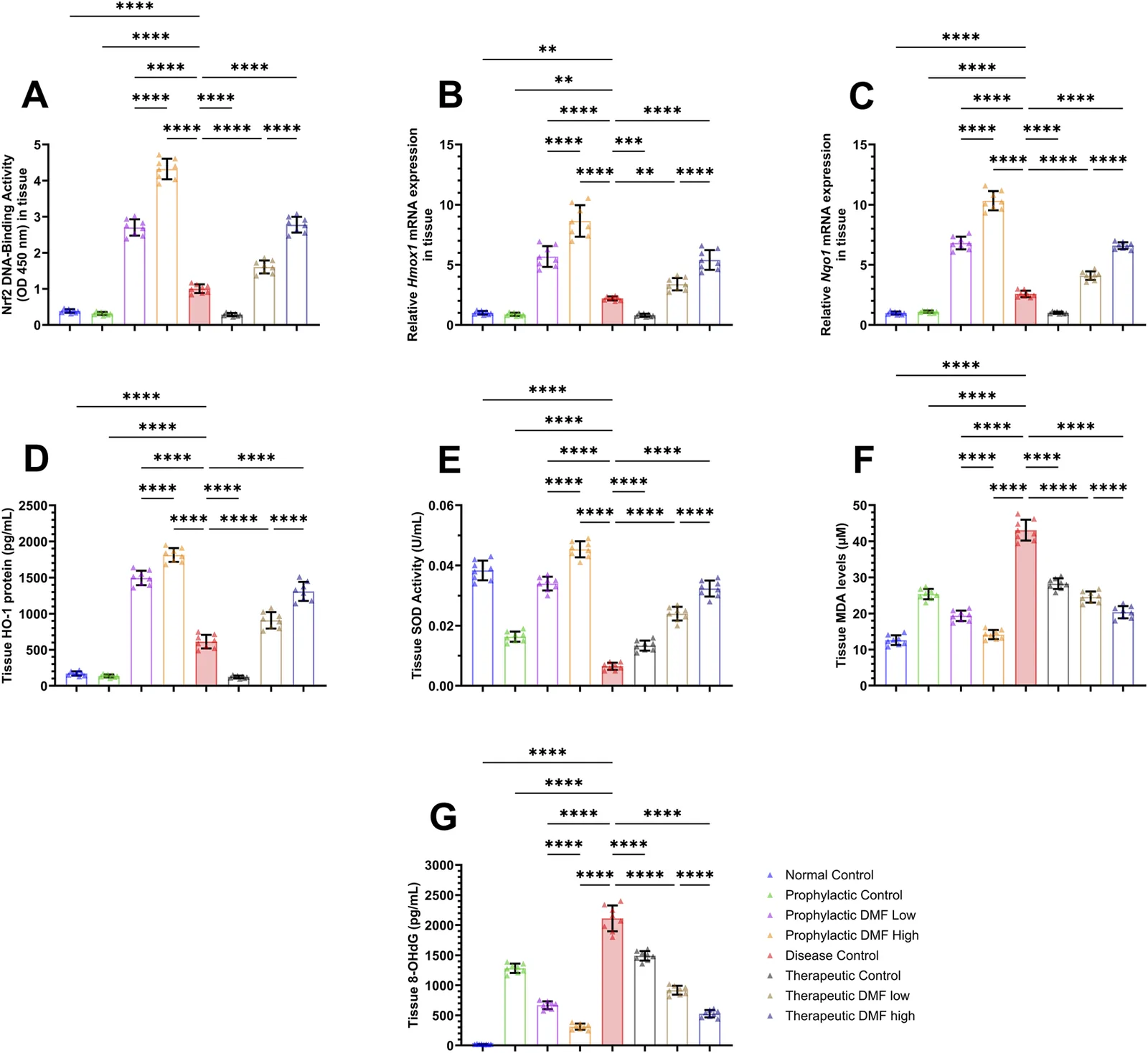
DMF activates the Nrf2/HO-1 antioxidant defense axis and mitigates structural oxidative damage. Effects of prophylactic and therapeutic DMF treatments on cellular defenses and oxidative injury markers in renal tissue. **(A)** Nrf2 DNA-binding activity measured via TransAM assay (OD 450 nm). Relative mRNA expression of downstream antioxidant targets: **(B)** Hmox1 and **(C)** Nqo1. **(D)** Tissue HO-1 protein concentration (pg/mL). **(E)** Endogenous Superoxide Dismutase (SOD) functional activity (U/mL). Assessment of oxidative structural degradation via **(F)** tissue Malondialdehyde (MDA) levels (µM) representing lipid peroxidation, and **(G)** tissue 8-OHdG levels (pg/mL) representing oxidative DNA damage. Data are presented as individual replicate values overlaid on mean ± SD (n = 8 subjects per group). Statistical variance was evaluated using a one-way ANOVA followed by Holm-Šídák’s multiple comparisons test. ****p < 0.0001, ***p < 0.001, and **p < 0.01 indicate significant differences between the specified groups.

Dose dependent activation of this antioxidant machinery was driven by DMF. The high-dose prophylactic DMF was a potent Nrf2 activator driving up Nrf2 DNA binding activity to 4.32 ± 0.28 (p < 0.0001 vs. Disease Control; Figure 5A). Such high transcriptional activation significantly regulated Hmox1 (8.65 ± 1.32-fold; p < 0.0001; Figure 5B) and Nqo1 (10.33 ± 0.79-fold; p < 0.0001; Figure 5C) and HO-1 translation (1811.88 ± 96.03 pg/mL; p < 0.0001) significantly increased. This robust activation of the Nrf2 pathway maintained and restored the enzymatic defenses and activity of SOD to normal levels (0.045 ± 0.003 U/mL; p < 0.0001; Figure 5E). The high-dose DMF also had a dose-dependent activation of this process (a major difference from the low-dose DMF).

### 3.5 Activation of the Nrf2/HO-1 antioxidant defense axis and mitigation of oxidative damage

To assess whether the antioxidant defense system was effective in keeping the renal tissue from reactive oxygen species, we measured structural oxidative damage. The collapse of endogenous defenses in the Disease Control group allowed for uncontrolled oxidative damage that was particularly high in lipid peroxidation and nucleic acid damage. The tissue MDA levels increased to 43.08 ± 2.91 µM from the normal physiological level of 12.60 ± 1.35 µM (p < 0.0001; Figure 5F). 8-OHdG, a key biomarker of oxidative DNA damage, rose to 2111.25 ± 214.51 pg/mL from a negligible baseline of 18.58 ± 5.03 pg/mL (p < 0.0001; Figure 5G).

The Nrf2/HO-1 shield provided by DMF worked to stop the oxidative assault. High dose prophylactic DMF was able to reduce lipid peroxidation to a very low level of MDA to 14.15 ± 1.27 µM, which is comparable to healthy controls (p < 0.0001 vs. Disease Control; Figure 5F). Structural DNA integrity was preserved with 8-OHdG accumulation limited to 313.13 ± 50.21 pg/mL (p < 0.0001; Figure 5G). The exposure to high dose therapy also yielded a significant reduction in oxidative damage with MDA to 20.38 ± 1.68 µM and 8-OHdG to 527.50 ± 60.77 pg/mL, which means that this drug does not contribute to further oxidative damage even after injury.

### 3.6 Inhibition of inflammasome priming and pyroptotic execution

To connect the oxidative stress profile with local inflammatory signaling, we investigated the transcriptional priming of the inflammasome complex. In the Disease Control group, the strong accumulation of cellular reactive oxygen species caused the critical oxidative sensor Txnip to surge up to 7.94 ± 0.88-fold higher than the 1.00 ± 0.11 normalized values of Normal Controls (p < 0.0001; Figure 6A). This oxidative activation in conjunction with NF-κB activation drove the transcription of the inflammasome structure, with Nlrp3 mRNA levels increasing to 8.79 ± 0.98-fold higher than normal physiological levels (0.98 ± 0.11, p < 0.0001; Figure 6B).

**FIGURE 6:**
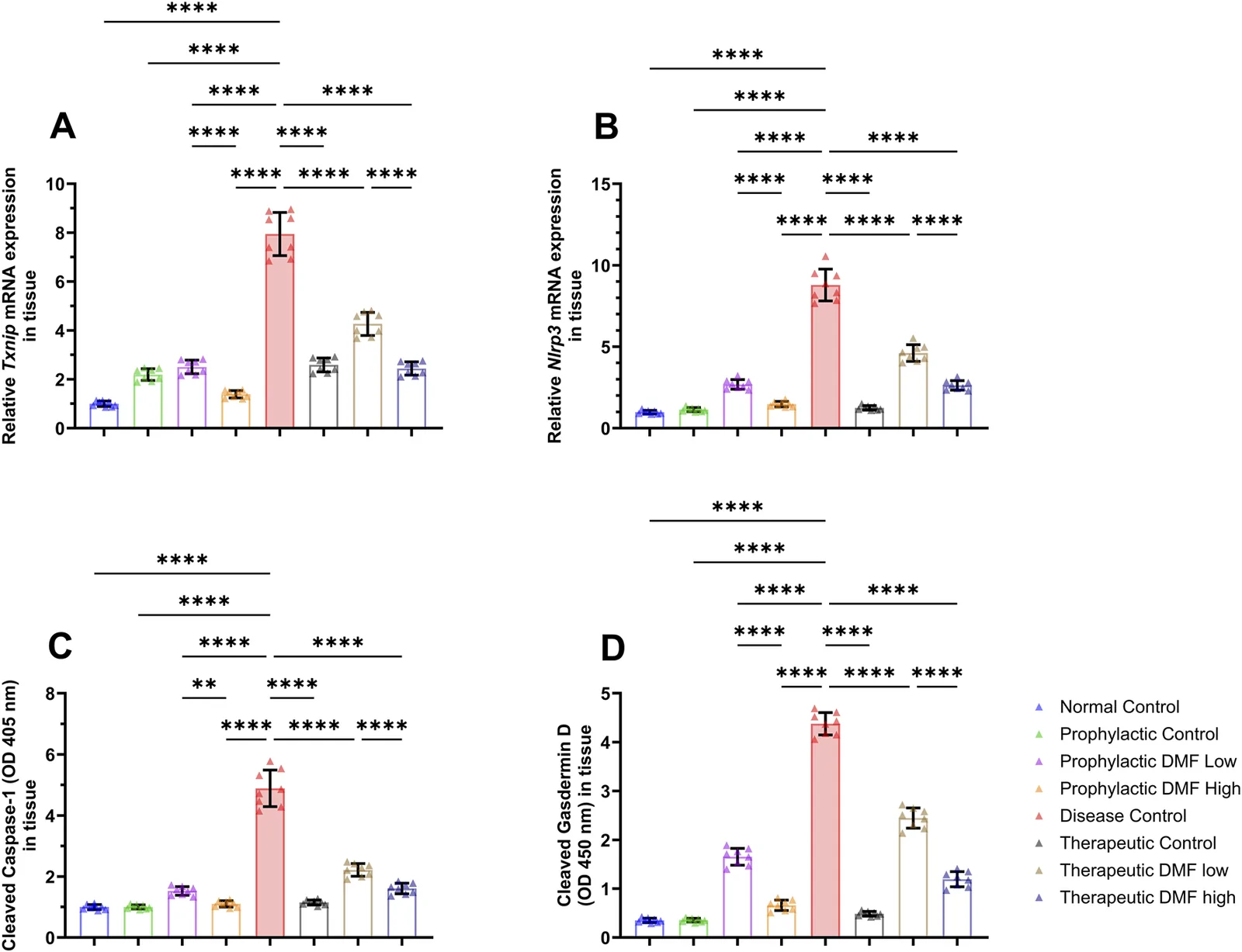
DMF inhibits inflammasome priming and pyroptotic execution. Effects of prophylactic and therapeutic treatments on the oxidative-inflammatory inflammasome axis in renal tissue. Transcriptional priming is represented by relative mRNA expression of **(A)** Txnip and **(B)** Nlrp3. Pyroptotic execution is assessed by measuring the functional activation of **(C)** Cleaved Caspase-1 via colorimetric enzymatic assay (OD 405 nm) and **(D)** Cleaved Gasdermin D via Sandwich ELISA (OD 450 nm). Data are presented as individual replicate values overlaid on mean ± SD (n = 8 subjects per group). Statistical variance was evaluated using a one-way ANOVA followed by Holm-Šídák’s multiple comparisons test. ****p < 0.0001 and p < 0.01 indicate significant differences between the specified groups.

By clearing oxidative burden and suppressing NF-κB signaling, DMF wiped out the inflammasome’s key mechanisms. High-dose DMF prevented the transcriptional priming phase from happening and was able to suppress the LPS-induced increase in Txnip mRNA to 1.39 ± 0.15-fold (p < 0.0001 vs. Disease Control; Figure 6A), and Nlrp3 mRNA transcription to 1.48 ± 0.16-fold (p < 0.0001; Figure 6B). By the same token, when DMF is applied in a dose-dependent manner, the inflammasome assembly is suppressed in a strong manner. As shown in Figure 6, high-dose DMF reduced Txnip (2.44 ± 0.27-fold) and Nlrp3 (2.62 ± 0.29-fold) expression significantly compared to Disease Control and the low-dose treatment (p < 0.0001), suggesting that DMF can suppress inflammasome priming even after taking it.

Following inflammasome assembly, we investigated the enzymatic activity of pyroptotic cell death by the NLRP3 inflammasome. In LPS-intoxicated subjects, the NLRP3 inflammasome was primed and oligomerized sufficiently to activate the main pyroptotic enzyme Cleaved Caspase-1 to 4.89 ± 0.60 from 1.00 ± 0.08 for Normal Controls (p < 0.0001; Figure 6C). The enzymatic activity then led to cleavage of the ultimate pyroptotic executioner, Cleaved Gasdermin D, in high levels (OD 4.38 ± 0.23 from 0.35 ± 0.04 in normal subjects and p < 0.0001; Figure 6D).

Because the upstream structural priming was completely eliminated by DMF, pyroptotic execution was stopped downstream. The high dose prophylactic treatment almost completely wiped out enzymatic activity of Cleaved Caspase-1 and Gasdermin D at baseline (1.10 ± 0.10, p < 0.0001 vs. Disease Control in Figure 6C). The final pore forming step was therefore blocked: Cleaved Gasdermin D accumulation was limited to 0.66 ± 0.11 (p < 0.0001 in Figure 6D). Moreover, DMF was effective in reducing Caspase-1 and Gasdermin D cleavage (p < 0.0001 for all comparisons against Disease Control). Thus, DMF successfully protects renal tissue from explosive and pyroptotic cell death.

### 3.7 Stabilization of mitochondrial integrity and apoptotic priming

In order to assess the effects of the treatments on programmed cell death, we also checked the mitochondrial apoptosis pathway by the transcriptional balance of the Bcl-2 protein family. In the Disease Control group, severe endotoxemia altered the mitochondrial survival thresholds dramatically. The primary pro-apoptotic channel-forming mediator, Bax, was up to 7.31 ± 0.86-fold higher than 1.00 ± 0.12 in normal subjects (p < 0.0001; Figure 7A), and transcription of the important anti-apoptotic guardian, Bcl2, was reduced to 0.35 ± 0.05-fold lower as compared to 1.02 ± 0.15 in normal controls (p < 0.0001; Figure 7B). As a result of this differential regulation, the pro-apoptotic Bax/Bcl2 survival ratio was increased to 21.64 ± 4.84, indicating a high level of mitochondrial stress and apoptotic priming (p < 0.0001 vs. 1.01 ± 0.22 in Normal Controls; Figure 7C).

**FIGURE 7:**
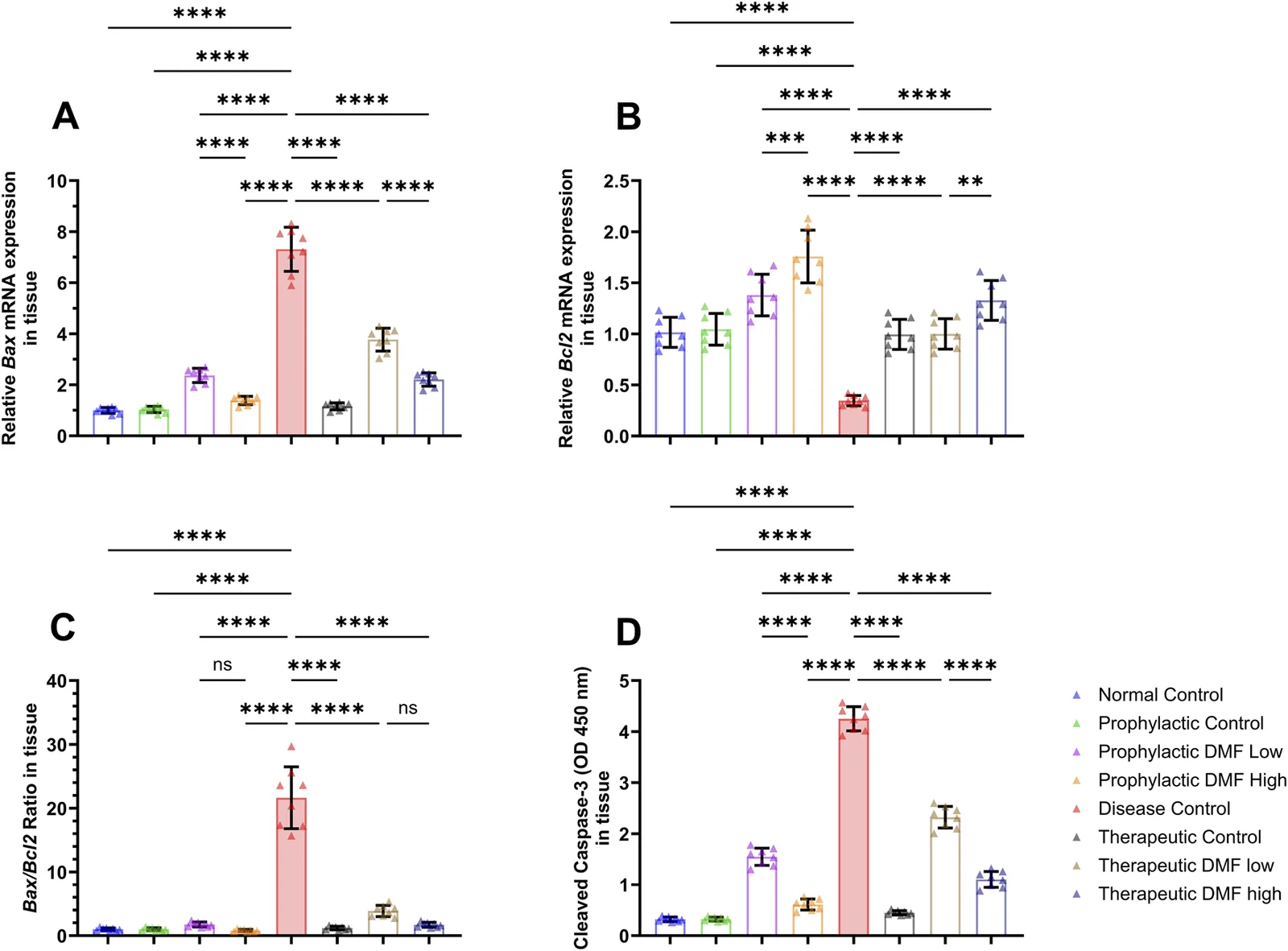
DMF stabilizes mitochondrial integrity and prevents apoptotic execution. Effects of prophylactic and therapeutic DMF treatments on the classical programmed cell death pathway in renal tissue. The mitochondrial stress threshold is represented by the relative mRNA expression of the pro-apoptotic marker **(A)** Bax and the anti-apoptotic guardian **(B)** Bcl2. **(C)** The calculated Bax/Bcl2 survival ratio demonstrates the balance of mitochondrial apoptotic priming. **(D)** Functional execution of apoptosis assessed via the enzymatic activation of Cleaved Caspase-3 (OD 450 nm). Data are presented as individual replicate values overlaid on mean ± SD (n = 8 subjects per group). Statistical variance was evaluated using a one-way ANOVA followed by Holm-Šídák’s multiple comparisons test. ****p < 0.0001, ***p < 0.001, and p < 0.01 indicate significant differences between the specified groups; ns indicates no significant difference (p > 0.05).

The intervention of DMF stabilized the mitochondrial axis and reversed this transcriptional imbalance. High-dose prophylactic DMF stopped the pro-apoptotic drive to 1.39 ± 0.16-fold (p < 0.0001 vs. Disease Control; Figure 7A) and at the same time promoted cellular survival by upregulating Bcl2 expression significantly above normal levels to 1.76 ± 0.26-fold (p < 0.0001). The Bax/Bcl2 ratio was therefore completely normalized in the high-dose prophylactic group (0.81 ± 0.18) and it was statistically better off for cellular survival compared to apoptosis (Figure 7C). The therapeutic DMF interventions also showed significant dose-dependent stabilization of the mitochondrial membrane potential and the high-dose treatment limited the Bax/Bcl2 ratio to 1.71 ± 0.38 (p < 0.0001 vs. Disease Control) which prevented apoptotic priming.

### 3.8 Prevention of apoptotic execution

After the mitochondrial instability, the final execution phase of the classical apoptotic cascade was measured in the main executioner protease. Due to the dysregulation of the upstream Bax/Bcl2, the LPS-intoxicated Disease Control group showed functional activation of Cleaved Caspase-3. At 4.25 ± 0.24 we see a large and unmitigated programmed cell death compared with the normal state of 0.32 ± 0.04 (p < 0.0001; Figure 7D).

Because DMF maintained mitochondrial integrity and eliminated oxidative triggers, the downstream terminal execution pathway was starved of its initiating signals. The high-dose prophylactic treatment effectively halted enzymatic execution and prevented Cleaved Caspase-3 activity to close to the baseline as well (0.61 ± 0.11, p < 0.0001 vs. Disease Control; Figure 7D). DMF also prevented further apoptotic execution by virtue of the dose-dependent effect. The high-dose treatment of DMF reduced Cleaved Caspase-3 to 1.10 ± 0.16, which was superior to the low-dose treatment (2.32 ± 0.21, p < 0.0001). Thus, DMF effectively halted renal apoptosis.

### 3.9 Amelioration of LPS-Induced renal histopathological alterations

To validate the biochemical and molecular evidence for renal protection, we assessed the gross microscopic structure of the kidney using semi-quantitative ATN scoring. For detailed morphological and visual measurements of individual nephron structures in all experiments (400x microscope), please refer to the supplementary document (Supplementary Material). The kidney tissue from the Normal Control group displays a typical, healthy histology, and the baseline ATN score was 0.09 ± 0.08 (Figure 8). The cortical structure is completely intact with regular glomerular tufts, clear Bowman’s capsules, and well-ordered proximal convoluted tubules (PCTs) with fuzzy brush borders.

**FIGURE 8:**
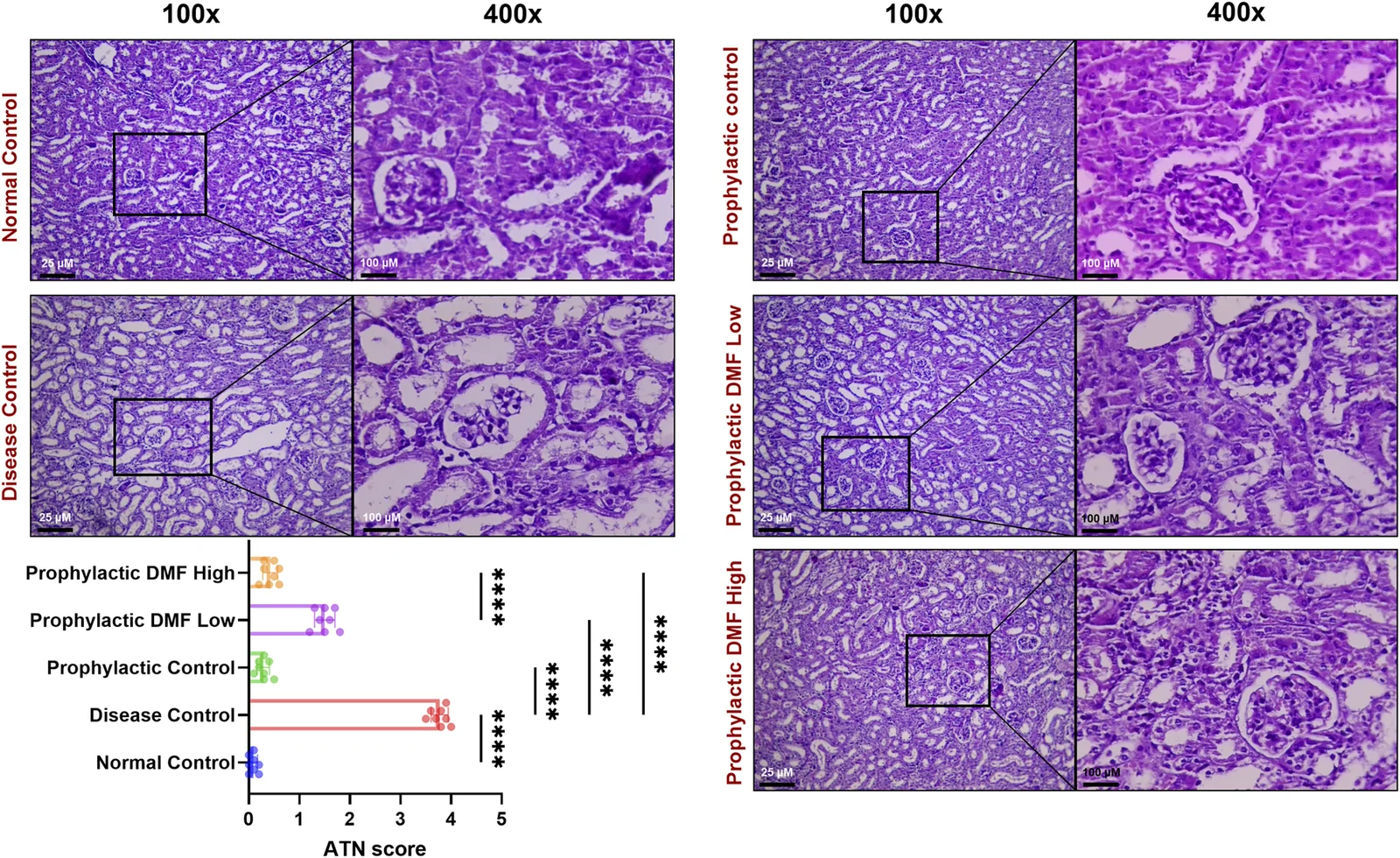
Prophylactic DMF prevents LPS-induced acute histopathological structural damage in renal tissue. Representative photomicrographs of renal cortical sections (Hematoxylin and Eosin stain; ×100 and ×400 magnification) alongside the corresponding semi-quantitative ATN injury scores. The Normal Control displays intact glomeruli and tubules with distinct brush borders, while the untreated Disease Control exhibits severe acute tubular necrosis. Prophylactic high-dose DMF heavily shields against tubular dilatation and epithelial desquamation, yielding protective scores comparable to the Dexamethasone Prophylactic Control. Bar graph data are presented as individual replicate values overlaid on mean ± SD (n = 8). Statistical variance was evaluated using a one-way ANOVA followed by Holm-Šídák’s multiple comparisons test. ****p < 0.0001 vs. the specified control group.

In contrast, LPS intoxication in the Disease Control group led to severe tubulointerstitial damage characteristic of acute tubular necrosis (ATN) and a very high mean ATN score of 3.78 ± 0.17. The cytoarchitecture was severely distorted and exhibited tubular dilatation, total loss of the apical brush borders, extensive interstitial inflammatory cell infiltration (mainly neutrophils and macrophages), and the presence of intraluminal proteinaceous necrotic casts (Figure 8).

DMF clearly protected renal cytoarchitecture (Figure 8). High-dose prophylactic DMF (100 mg/kg) provided strong structural protection and limited the ATN score to 0.43 ± 0.15. The tissue was mostly intact with intact tubular linings and separate brush borders, and the interstitial inflammatory infiltration was almost nonexistent, as was the Dexamethasone Prophylactic Control (0.29 ± 0.12). The low-dose prophylactic treatment (50 mg/kg) also provided visible protection (ATN score: 1.50 ± 0.20) but showed moderate tubular swelling and partial loss of the delicate apical brush borders (Figure 8).

When administered therapeutically after the LPS-induced cytokine storm started taking hold, DMF was able to alleviate the structural degradation (Figure 9). As such, the low-dose group (50 mg/kg) had partial histological rescue (ATN score: 2.15 ± 0.24) and it successfully prevented total terminal desquamation, but still showed signs of epithelial swelling, luminal dilatation, and early osmotic stress. However, the high-dose group (100 mg/kg) had a complete restoration of structural integrity and the ATN score was reduced to 0.85 ± 0.16. The glomerular tuft of the high-dose treatment group showed strong preservation, tight epithelial lining, and a complete clearance of intraluminal casts and heavy inflammatory infiltrates and showed better clinical outcomes (ATN score: 0.56 ± 0.14) (Figure 9).

**FIGURE 9:**
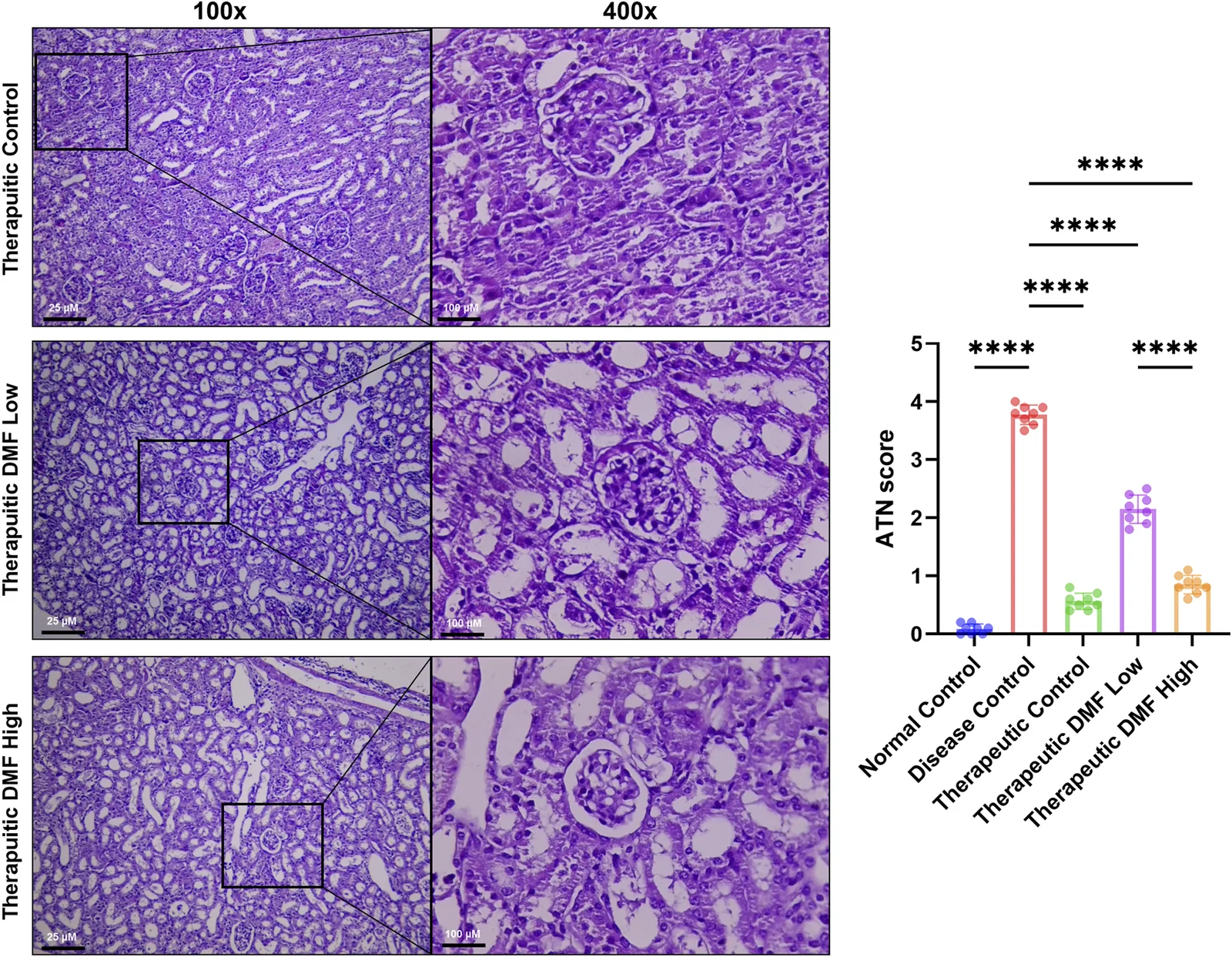
Therapeutic DMF halts and mitigates ongoing LPS-induced acute tubular necrosis. Representative photomicrographs of renal cortical sections (Hematoxylin and Eosin stain; ×100 and ×400 magnification) and the corresponding semi-quantitative ATN injury scores. When administered post-LPS challenge, high-dose therapeutic DMF forcefully limits terminal cellular sloughing, clears intraluminal necrotic debris, and suppresses interstitial inflammation, matching the rescue efficacy of the Dexamethasone Therapeutic Control. Bar graph data are presented as individual replicate values overlaid on mean ± SD (n = 8). Statistical variance was evaluated using a one-way ANOVA followed by Holm-Šídák’s multiple comparisons test. ****p < 0.0001 vs. the specified control group.

## 4 Discussion

The pathogenesis of endotoxemia-associated acute kidney injury is a serious clinical problem due to an intricate interplay of cytokine release syndrome, unchecked oxidative stress and programmed cellular death. Though a single high-dose LPS challenge does not fully capture the infectious and progressive hemodynamics of clinical human sepsis, it is a good model for isolating the acute, systemic hyperinflammatory phase of endotoxic shock. Current clinical management is largely reactive and supportive. In this study, we have shown that Dimethyl fumarate, an FDA-approved immunomodulatory agent, can minimize lipopolysaccharide-induced acute kidney injury due to its potent and dual-axis modulation of TLR4/NF-κB and Nrf2/HO-1 signaling pathways. Our results have demonstrated that timing is very important to treatment and are a good basis for translating Dimethyl fumarate to acute critical care nephrology (Figure 10).

**FIGURE 10:**
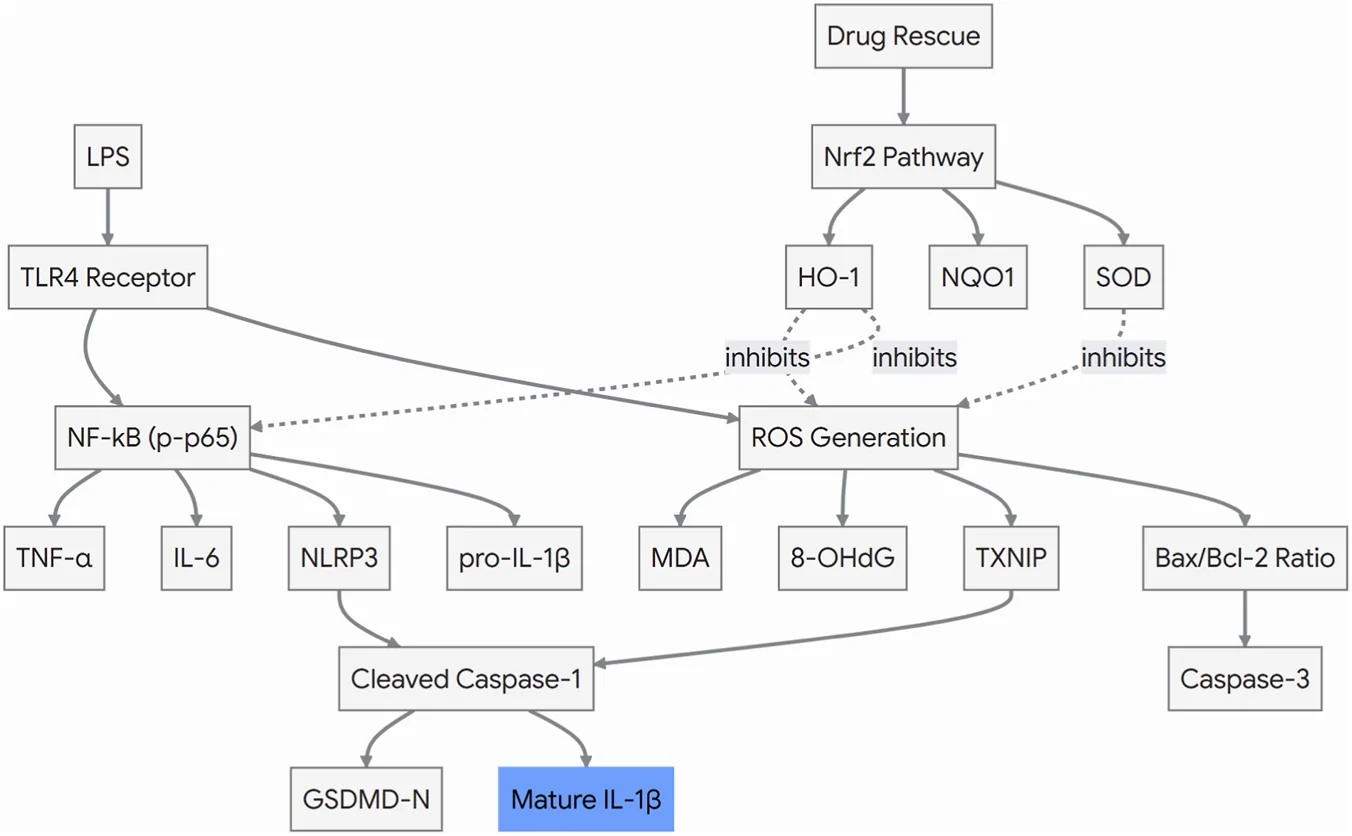
Molecular Pathway Crosstalk in AKI and Mechanisms of DMF Rescue. The schematic illustrates the dual-axis protection afforded by Dimethyl Fumarate (DMF) during lipopolysaccharide (LPS)-induced acute kidney injury. The inflammatory pathogenesis begins with LPS binding to the TLR4 receptor, which concurrently triggers massive Reactive Oxygen Species (ROS) generation and flips the master inflammatory switch via nuclear translocation of NF-κB (p-p65). This unmitigated NF-κB activity drives systemic inflammation (TNF-α, IL-6) and provides “Signal 1”(NLRP3, pro-IL-1β) for inflammasome assembly. Simultaneously, the oxidative storm shreds cellular membranes (MDA), damages DNA (8-OHdG), shifts the mitochondrial survival threshold toward apoptosis (Bax/Bcl-2 Ratio), and provides “Signal 2”(TXNIP). The convergence of NLRP3 and TXNIP triggers pyroptosis execution via Cleaved Caspase-1, culminating in lethal membrane pore formation (GSDMD-N) and the release of Mature IL-1β. DMF intervention establishes a profound cross-talk blockade by forcefully upregulating the Nrf2 defense pathway. The resultant generation of HO-1 physically inhibits NF-κB activation, successfully silencing inflammatory priming, while the restoration of SOD neutralizes ROS generation. By starving the downstream cascades of both inflammatory and oxidative triggers, DMF aborts the execution of Caspase-1-mediated pyroptosis and Caspase-3-mediated apoptosis, thereby preventing the structural collapse of the renal tissue.

The robust cytoprotective effects of Dimethyl fumarate observed in this study are driven by the profound disruption of the intricate molecular cross-talk between the TLR4/NF-κB inflammatory cascade, the Nrf2/HO-1 antioxidant defense system, and the terminal execution of programmed cell death. In the pathogenesis of sepsis-associated acute kidney injury, the initial insult begins at the cell surface where lipopolysaccharide binds to the TLR4 receptor, triggering the primary inflammatory transcription factor to enter the nucleus (Zhou et al., 2021). This upregulates TLR4 mRNA in a pathological feed-forward loop and increases Phospho-NF-κB p65, effectively flipping the master inflammatory switch that drives the massive transcription and systemic release of TNF-α and IL-6 (Fang et al., 2023). By significantly suppressing both TLR4 and Phospho-p65, prophylactic Dimethyl fumarate successfully silences this master switch before it can prime downstream destructive pathways (Yang et al., 2021).

Concurrently, lipopolysaccharide induces a massive oxidative storm that rapidly exhausts endogenous defenses, causing Superoxide Dismutase activity to plummet. This allows reactive oxygen species to severely damage cellular structures, shredding cell membranes and damaging nuclear DNA, as evidenced by elevated malondialdehyde and 8-OHdG levels. Dimethyl fumarate forcefully counters this cascade by causing Nrf2 DNA-binding activity to surge, which forces the transcription of Hmox1 and Nqo1 (Gao et al., 2024), ultimately restoring Superoxide Dismutase activity and generating massive amounts of HO-1 protein (Yang et al., 2021). The generation of HO-1 represents a critical cross-talk checkpoint between the antioxidant and inflammatory axes; the HO-1 protein produces physiological byproducts that physically inhibit NF-κB (Thomas et al., 2022). The simultaneous presentation of high Nrf2/HO-1 activity alongside low Phospho-p65 in our treated cohorts confirms that the augmented antioxidant pathway actively cross-talks to shut down the inflammatory axis.

This dual-mechanism fundamentally converges at the execution of pyroptosis, which strictly requires two distinct signals to trigger explosive cell death. The first priming signal is driven by NF-κB, which spikes the transcription of Nlrp3 and pro-Il1b to build the raw structural materials of the inflammasome (Hussein et al., 2024). The second triggering signal is driven by reactive oxygen species, which activate the critical oxidative sensor TXNIP. During unmitigated injury, TXNIP binds to NLRP3, activating Cleaved Caspase-1, which subsequently cuts Gasdermin D to open lethal membrane pores and release mature IL-1β into the systemic circulation (Zhou et al., 2024). Because Dimethyl fumarate clears reactive oxygen species (thereby lowering TXNIP) and directly blocks NF-κB (thereby lowering NLRP3), the inflammasome complex is starved of both its oxidative trigger and its transcriptional building blocks (Li et al., 2022), effectively aborting the cleavage of Caspase-1 and Gasdermin D.

Furthermore, the combination of intense oxidative stress and high TNF-α levels triggers silent, programmed apoptotic cell death alongside pyroptosis. This extreme mitochondrial stress shifts the critical survival threshold by upregulating pro-apoptotic Bax mRNA and downregulating anti-apoptotic Bcl2 mRNA, ultimately leading to the cleavage of the final executioner, Caspase-3 (Li et al., 2024). By utilizing the Nrf2/HO-1 axis to clear reactive oxygen species and halt upstream lipid and DNA damage (Yang et al., 2021), Dimethyl fumarate actively normalizes the Bax/Bcl2 ratio and halts Caspase-3 cleavage (Gao et al., 2024).

Ultimately, the unmitigated culmination of systemic inflammation, pyroptosis, and apoptosis results in gross structural and functional organ collapse. As tubular cells die, they release KIM-1 into the surrounding tissue, leading to a catastrophic loss of filtration capacity and corresponding elevations in serum BUN and creatinine (Yang et al., 2025). The definitive preservation of BUN, creatinine, and KIM-1 in the drug-treated cohorts serves as the final *in vivo* proof that simultaneously silencing the upstream TLR4/NF-κB and reactive oxygen species pathways successfully prevents structural organ collapse (Li et al., 2024; Zhou et al., 2021).

The gross morphological changes observed in the renal cytoarchitecture serve as the definitive *in vivo* proof of concept for this study, directly translating the subcellular molecular signaling cascades into macroscopic structural outcomes. The semi-quantitative Acute Tubular Necrosis scoring perfectly mirrors the biochemical and transcriptional data, validating that the dual-axis molecular modulation provided by Dimethyl fumarate physically preserves the functional architecture of the nephron.

At the molecular level, lipopolysaccharide binding to TLR4 triggers the rapid nuclear translocation of NF-κB, unleashing a massive transcriptional wave of pro-inflammatory cytokines, including TNF-α and IL-6. This subcellular biochemical signal acts as a potent systemic and local chemotactic gradient (Sanz et al., 2010). Histologically, this gradient physically manifests in the untreated Disease Control cohort as severe interstitial inflammatory cell infiltration, characterized by dense swarms of neutrophils and macrophages invading the spaces between the tubules (Zhou et al., 2021). The striking clearance of these immune products in the interstitium of the Dimethyl fumarate-treated cohorts provides direct visual proof that suppressing Phospho-p65 upstream successfully abolishes the chemotactic recruitment of inflammatory cells into the renal cortex (Fang et al., 2023).

The unmitigated oxidative storm, marked by depleted Superoxide Dismutase and soaring malondialdehyde and 8-OHdG levels, rapidly destabilizes the mitochondrial membrane, shifting the Bax/Bcl2 survival ratio and activating Caspase-3 (Li et al., 2024). Macroscopically, this programmed cellular death and lipid membrane peroxidation translate to the physical destruction of the proximal convoluted tubules. The Disease Control tissues exhibit severe cellular attenuation, widespread luminal dilatation due to osmotic stress, and the total effacement of the delicate apical brush borders (Baker and Cantley, 2025; Yang et al., 2021). By potently activating the Nrf2/HO-1 antioxidant shield, Dimethyl fumarate chemically neutralizes the reactive oxygen species before they can shred the lipid membranes. The histological proof of this molecular rescue is the preservation of thick, highly eosinophilic tubular walls and the maintenance of intact, fuzzy brush borders in the high-dose prophylactic and therapeutic cohorts (Yang et al., 2021).

The terminal execution of Gasdermin D-mediated pyroptosis fundamentally requires the rupture of the cellular membrane. When thousands of tubular epithelial cells undergo this explosive, highly inflammatory death simultaneously, they completely detach from the basement membrane and aggregate within the nephron. Histologically, this is visualized as the dense, proteinaceous necrotic casts that physically plug the distal and proximal tubules in the untreated subjects (Baker and Cantley, 2025). Because Dimethyl fumarate starves the NLRP3 inflammasome of its essential priming by NF-κB and triggering by TXNIP/ROS signals, the cleavage of Gasdermin D is aborted (Li et al., 2022). The definitive morphological proof of this aborted pyroptosis is the absence of intraluminal casts; the Dimethyl fumarate-treated tubules feature clear, unobstructed lumens and epithelial linings that remain tightly anchored to their basement membranes (Li et al., 2024).

Ultimately, the structural disparities between the dosing regimens visually validate the molecular timeline. The prophylactic Dimethyl fumarate regimen preemptively established an Nrf2/HO-1 shield and locked the NF-κB switch, resulting in near-perfect architectural preservation that rivaled healthy controls. Conversely, the high-dose therapeutic regimen was administered after the inflammatory and oxidative triggers were already pulled; however, its ability to yield structurally sound, cast-free tubules with significantly reduced swelling serves as the ultimate proof of concept that Dimethyl fumarate can actively intercept and halt ongoing apoptotic and pyroptotic execution even during an active cytokine storm.

A critical pharmacological divergence observed in this study is the marked difference in the anti-inflammatory efficacy between the prophylactic and therapeutic regimens. While prophylactic pre-loading of Dimethyl fumarate yielded a strict, dose-dependent suppression of the TLR4/NF-κB axis and systemic cytokines, therapeutic administration exhibited a distinct anti-inflammatory “ceiling effect.” When administered one hour post-intoxication, both the low and high therapeutic doses suppressed NF-κB activation, TNF-α, and IL-6 to statistically comparable levels. This indicates that the capacity of the drug to suppress the inflammatory cascade saturates at the lower dosage in a post-insult setting.

This ceiling effect is mechanistically tied to the hyperacute kinetics of lipopolysaccharide-induced endotoxemia. By the time the therapeutic intervention is administered at one hour post-challenge, the initial pathological binding of lipopolysaccharide to TLR4 has already occurred, and the first massive wave of NF-κB nuclear translocation and cytokine transcription has been irreversibly initiated (Covert et al., 2005). Consequently, therapeutic Dimethyl fumarate can actively halt ongoing and subsequent inflammatory signaling, but it cannot retroactively undo the initial transcriptional burst (Zhang et al., 2023). Thus, the lower therapeutic dose is sufficient to achieve the maximum possible post-insult inflammatory blockade, rendering higher doses redundant for this specific anti-inflammatory pathway (Fang et al., 2023).

However, despite reaching this inflammatory ceiling, the high-dose therapeutic regimen provided vastly superior structural preservation, normalized the apoptotic survival ratio, and significantly reduced functional markers of kidney injury (BUN, creatinine, and KIM-1) compared to the low-dose regimen (Yang et al., 2021). This disconnect definitively proves that the superior rescue capacity of high-dose therapeutic Dimethyl fumarate (Zhou et al., 2021) is not driven by increased anti-inflammatory action, but rather by its potent, dose-dependent antioxidant activity (Gao et al., 2024).

Once the cytokine storm has been triggered, the primary mechanistic advantage of the drug shifts from preemptive NF-κB suppression to aggressive Nrf2/HO-1 activation. The higher therapeutic dose is strictly required to generate sufficient HO-1 and restore enough Superoxide Dismutase to actively scavenge the massive, already-accumulating burden of reactive oxygen species. By forcefully clearing this established oxidative storm, high-dose therapeutic Dimethyl fumarate neutralizes the secondary oxidative triggers (TXNIP) required for inflammasome execution and prevents ROS-induced mitochondrial collapse. Therefore, in a therapeutic setting, the drug relies predominantly on its potent antioxidant capacity to halt terminal cellular necrosis and rescue the organ, effectively overriding the ongoing inflammatory damage.

From a translational perspective, defining the exact clinical applications for these distinct regimens is critical. While the unpredictable onset of community-acquired sepsis precludes targeted prophylactic preloading, the near-absolute structural shielding achieved by the 3-day prophylactic regimen is clinically relevant for anticipated inflammatory events. Specifically, high-risk patients scheduled for major elective gastrointestinal surgeries (which carry a high risk of bowel ischemia and endotoxin translocation), cardiopulmonary bypass operations (which predictably induce severe systemic inflammatory response syndrome), or planned oncological treatments known to trigger cytokine release syndrome (such as CAR-T cell therapy) represent ideal clinical populations for DMF pre-conditioning. In these controlled scenarios, establishing an upregulated Nrf2/HO-1 antioxidant shield prior to the anticipated insult could proactively prevent endotoxemia-associated AKI.

### 4.1 Study strengths and limitations

#### 4.1.1 Strengths

The main strength of this study is the fact that this study is highly translatable and has multiple levels. By comparing pre- and post-insult prophylactic and active therapeutic protocols, we distinguish between preventative tissue protection and active pharmacological rescue, and this study offers valuable information in a more complex clinical situation such as sepsis. Furthermore, drug dosages were calculated in accordance with FDA-standardized Body Surface Area allometric scaling for the murine, which is precisely comparable to the clinically known HED of Dimethyl fumarate. The study also provides a clear proof of concept by including the subcellular molecular cross-talk (TLR4/NF-κB and Nrf2/HO-1) as well as the final terminal outcomes (programmed cell death (pyroptosis and apoptosis), macroscopic structural preservation (histopathology), and functional organ capacity (BUN, creatinine, and KIM-1).

#### 4.1.2 Limitations

However, there are several limitations to these results. First, although the lipopolysaccharide-induced endotoxemia model is a well-established and reliable model to simulate the acute cytokine storm and oxidative stress of sepsis, it is not a hemodynamic instability model and polymicrobial complexity model of clinical human sepsis. Second, the molecular and histological data at a single terminal point is a clear snapshot of organ injury and can camouflage the early temporal dynamics of rapidly changing inflammatory sensors and occasional apoptotic markers. At the same time, because a lot of subjects died of endotoxic shock before the 6-h mark and therefore were not given sufficient samples for an accurate biochemical analysis, the untreated Disease Control group will be survivor-biased. The molecular and structural damage reported in the unmitigated disease state probably underestimate the true damage of the disease in this case since the most vulnerable animals were not included in the final quantitative biochemical analysis. The main limitation of the design for our study is the timing of the treatment and the cumulative drug exposure. Prophylactic patients were given repeated doses for 3 days to achieve systemic pre-loading before the endotoxic challenge, and therapeutic patients had only one dose after insult, and hence the differences in the drug effects were not necessarily due to the timing of the treatment. In addition, the prophylactic animals had much higher cumulative drug exposure and had already been saturated with tissue, which is why they are better protected against the endotoxic threat. Also, the study is focused on the localized kidney cytopathology, and the effect of Dimethyl fumarate on systemic organ cross-talk (cardiorenal or hepatorenal interaction) of the kidney in acute kidney injury needs to be studied in a more general systemic context. Finally, we only studied male mice in order to avoid hormonal variations around the estrous cycle. However, biological sex in acute inflammatory response is an established variable, and females are generally more robust to endotoxemia and AKI due to estrogen-mediated protective mechanisms. DMF is currently widely used and effective in female-dominated diseases, such as multiple sclerosis, but future work should consider female patients in order to determine if sex-dependent hormonal profiles affect the timing-dependent efficacy and therapeutic threshold of DMF in acute endotoxic shock.

## 5 Conclusion

Dimethyl fumarate has a very strong dose-dependent cytoprotective effect on sepsis-associated acute kidney injury by disrupting the destructive molecular cross-talk between systemic inflammation and oxidative stress. By upregulating the Nrf2/HO-1 antioxidant defense system and blocking the TLR4/NF-κB inflammatory master switch, Dimethyl fumarate can starve the NLRP3 inflammasome of its signals of priming and triggering as well as maintain mitochondrial membrane integrity. This dual-axis action prevents Gasdermin D-mediated pyroptosis and Caspase-3-mediated apoptosis and prevents the renal tubules from completely collapsing and the filtration system from being compromised. The key difference is in the timing of the intervention; while prophylactic treatment is a near-complete protection of the renal structure, high-dose treatment is required to prevent and halt an oxidative storm from occurring and rescue the organ even after the inflammatory cascade has begun. Because these protective mechanisms were achieved using dosages that directly translate to safely tolerated human limits, these findings provide a compelling, evidence-based rationale for the rapid clinical repurposing of Dimethyl fumarate as a viable mechanism-driven adjunctive therapy in critical care nephrology.

## Data Availability

The datasets presented in this study can be found in online repositories. The names of the repository/repositories and accession number(s) can be found below: https://doi.org/10.5281/zenodo.21908130.
