# Supplementary material for: Timing dictates efficacy: evaluating the prophylactic and therapeutic potential of dimethyl fumarate against LPS-induced shock
Source: Front Pharmacol. 2026 Oct 1;17:1966178. doi: 10.3389/fphar.2026.1966178 (PMC13669161; doi:10.3389/fphar.2026.1966178)
Supplement: Supplementary file 1 [file DataSheet1.pdf]

Supplementary Material

To enhance transparency and explicitly map the visual data to our injury scoring criteria, the legends below detail how the isolated high-magnification fields correspond to the specific hallmarks of Acute Tubular Necrosis (ATN) evaluated in this study.

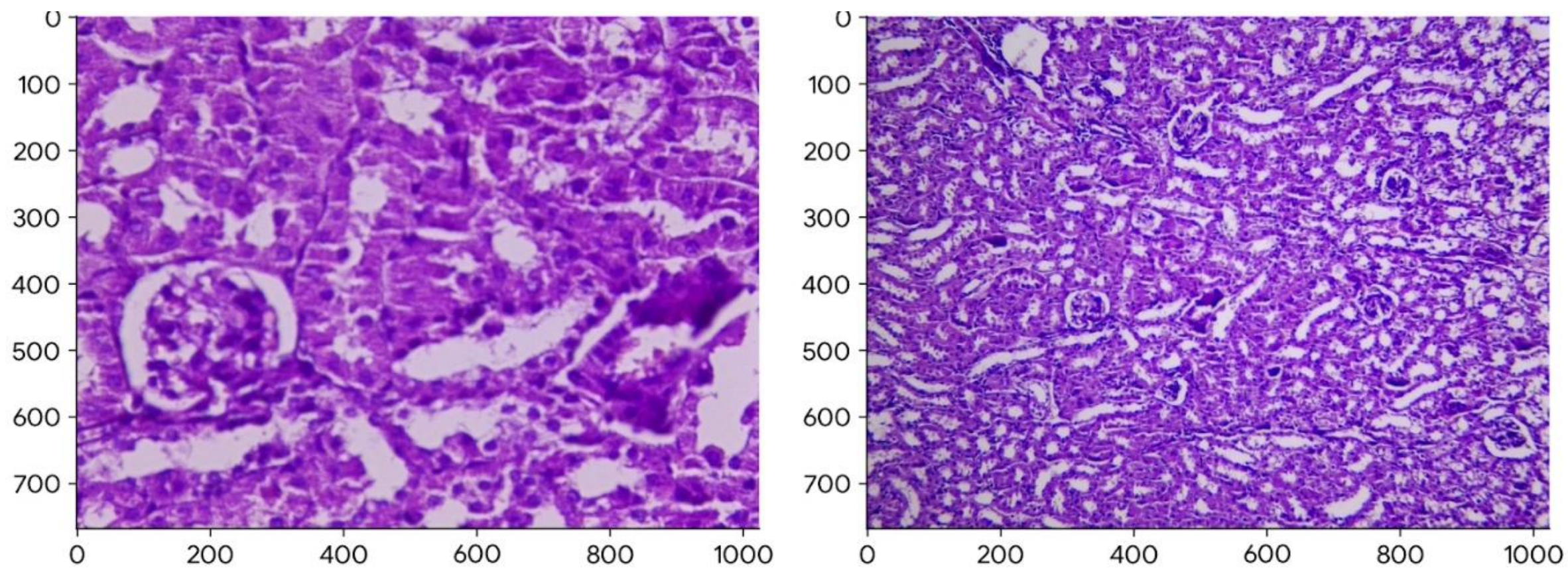

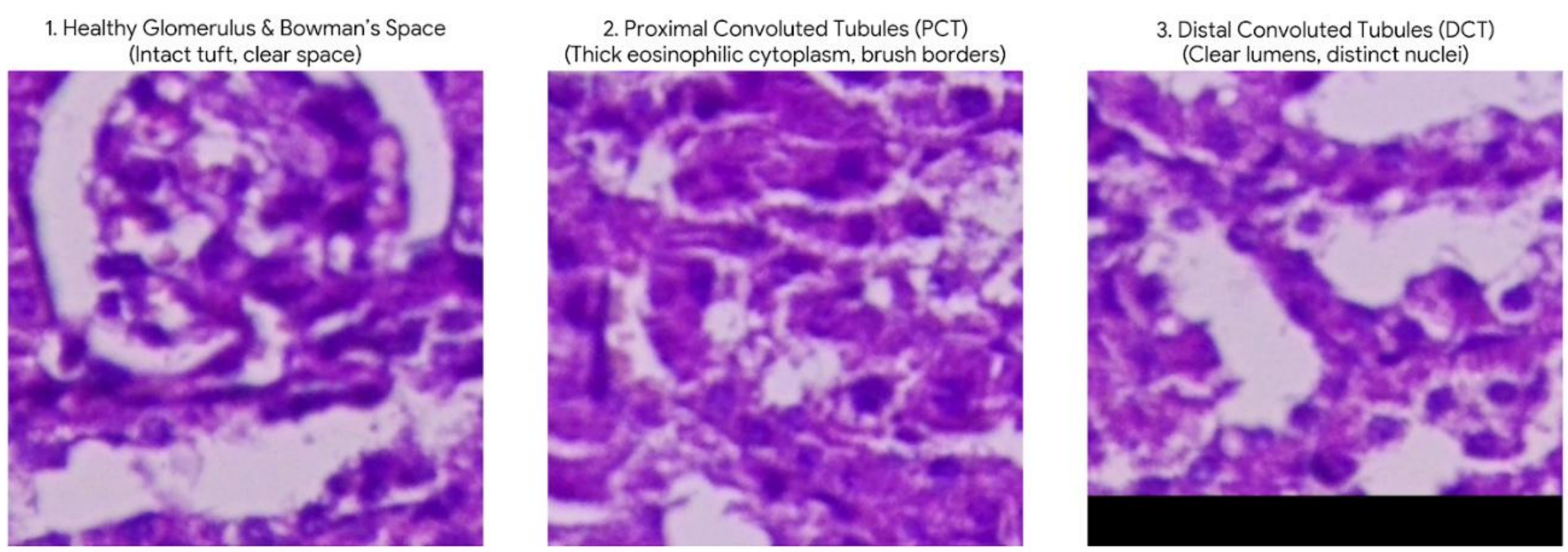

Supplementary Figure S1: Isolated Histological Features of healthy control (400x Magnification)

1. **Healthy Glomerulus & Bowman's Space (Left Panel):** This crop isolates the renal corpuscle. The dark, densely packed capillary tuft of the glomerulus is clearly visible. Surrounding it is the clear, unobstructed white crescent of Bowman's space, indicating no inflammation or crescentic formations.

2. **Proximal Convoluted Tubules (Middle Panel):** This isolates the PCTs, which perform the heavy lifting in the kidney. They are characterized by thick, highly eosinophilic (bright pink) cellular walls. Notice how the inner edges facing the white lumen appear slightly fuzzy or thick—this is the healthy, intact brush border.

3. **Distal Convoluted Tubules (Right Panel):** This isolates the DCTs. Compared with the PCTs, these tubules have thinner, less intensely pink cellular walls and much wider, clearer lumens. The nuclei (dark purple dots) also appear more closely packed around the ring.

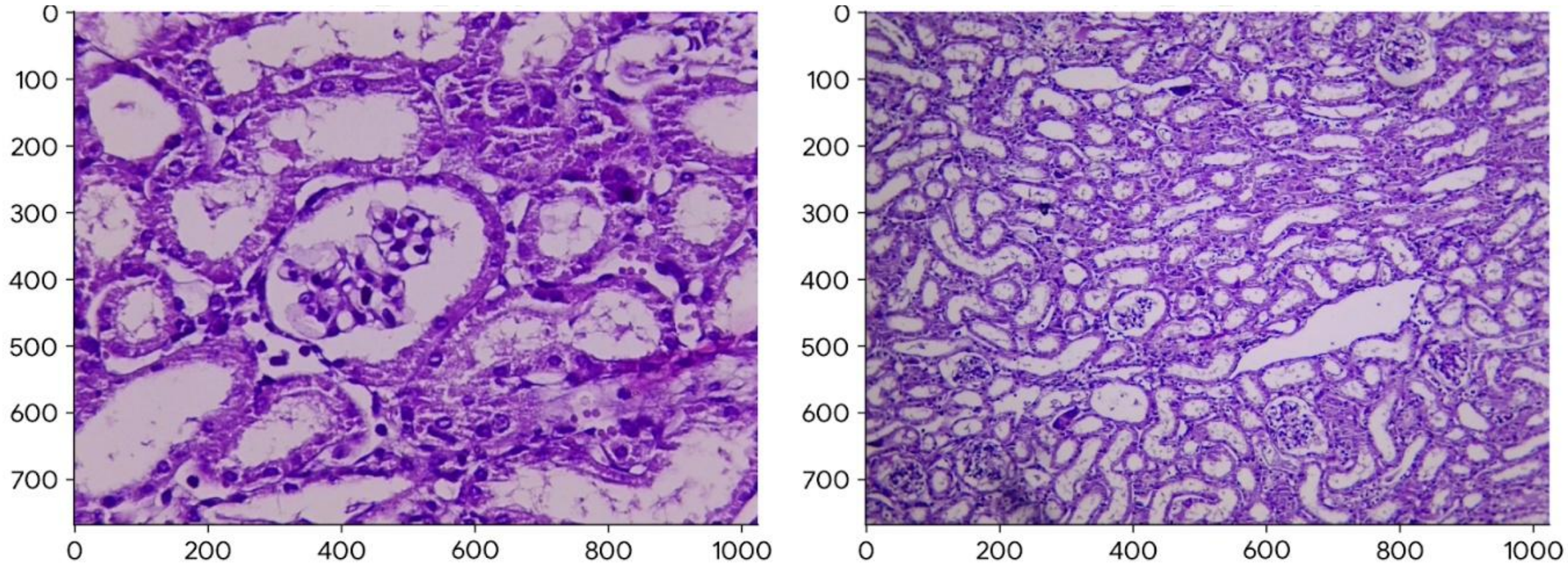

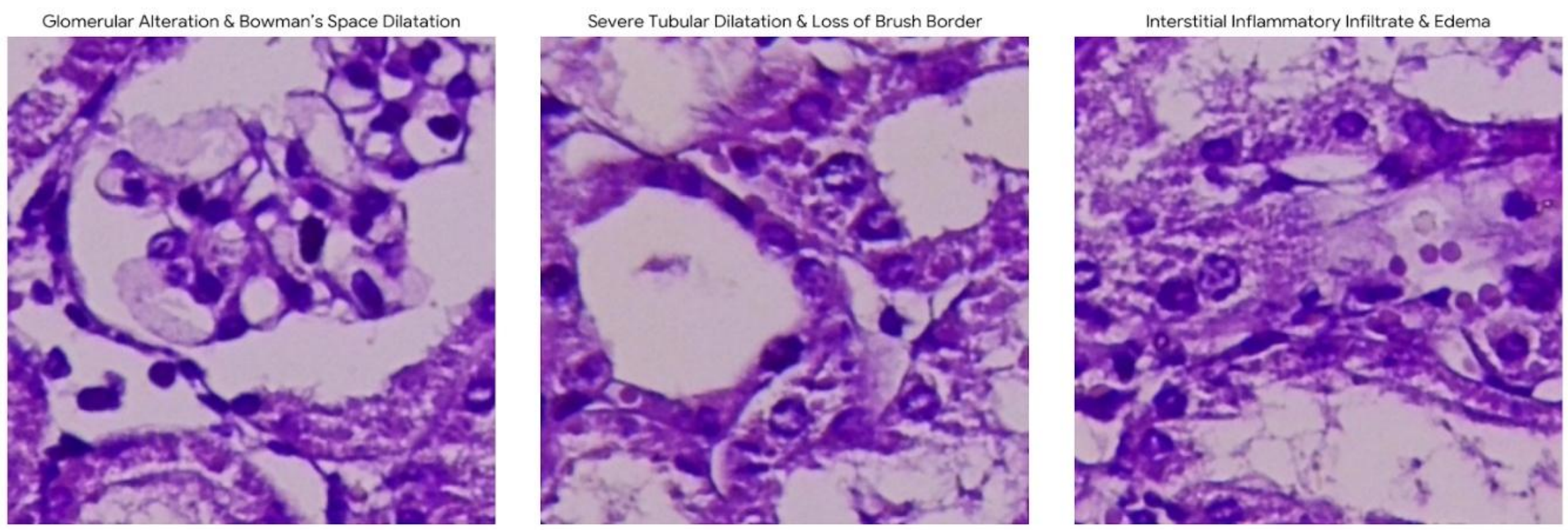

Supplementary Figure S2: Isolated Pathological Features of Disease Control (400x Magnification)

1. **Glomerular Alteration & Bowman's Space Dilatation (Left Panel):** Unlike the healthy, plump glomerulus seen in the normal control, this capillary tuft has shrunk and collapsed inward. Consequently, Bowman's space has widened markedly. You can also see faint, pale-pink proteinaceous fluid leaking into the space due to a compromised filtration barrier.

2. **Severe Tubular Dilatation & Loss of Brush Border (Middle Panel):** This isolates a severely damaged Proximal Convoluted Tubule, directly illustrating three key criteria of our ATN scoring system: tubular dilation, flattening of the tubular epithelium, and loss of the brush border. The lumen has expanded dramatically (the large white void). Notice how the inner cellular edges are entirely flat, sharp, and bare—the fuzzy, protective brush border has been completely destroyed. Furthermore, the epithelial cells have flattened (attenuated) as they struggle to stretch around the swollen tubule.

3. **Interstitial Inflammatory Infiltrate & Edema (Right Panel):** In the spaces between the tubules, you can see numerous small, dark-purple, densely stained nuclei. These are clusters of inflammatory cells (primarily neutrophils and macrophages) invading the tissue in response to the LPS-induced cytokine storm, fulfilling the 'interstitial inflammatory cell infiltration' metric of the ATN score. The large white gaps around these cells represent severe interstitial edema (fluid swelling that separates the tissue layers).

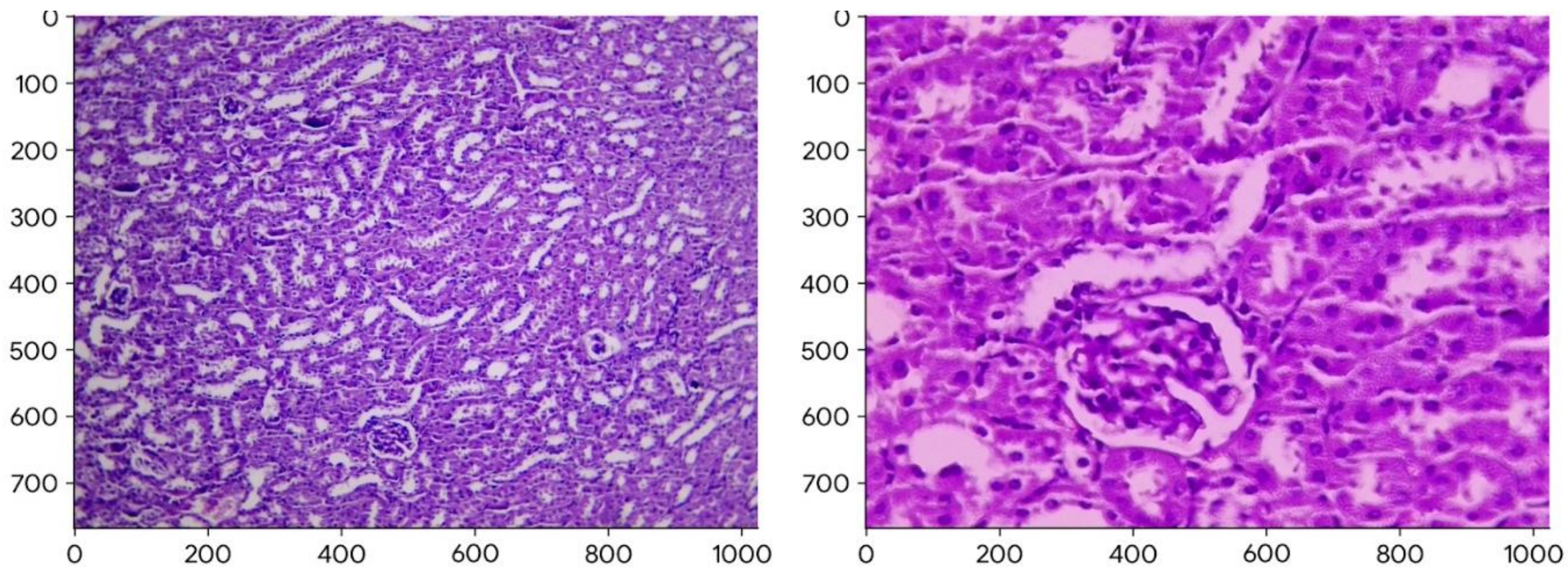

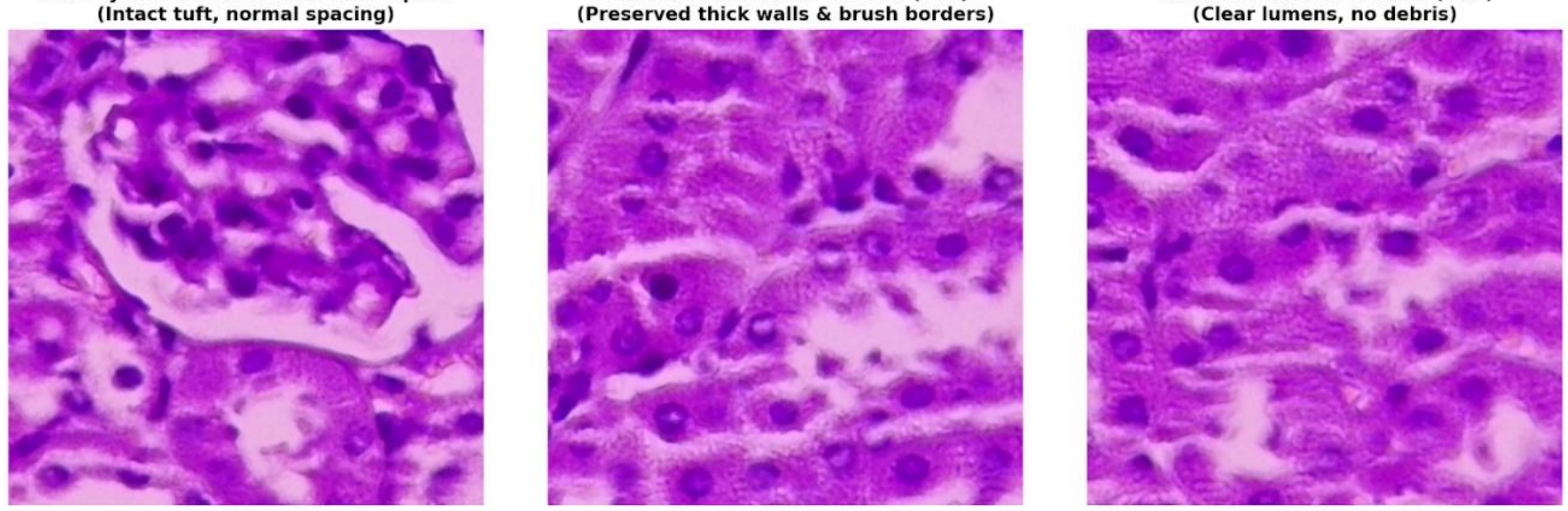

Supplementary Figure S3: Isolated Pathological Features of Prophylactic Control (400x Magnification)

At the cellular level, the nephron structures are fully preserved. I have isolated the three key features from your 400x image to match the format of the previous panels.

1. **Healthy Glomerulus & Bowman's Space (Left Panel):** The glomerular capillary tuft is prominent, plump, and healthy. It has not collapsed, and Bowman's space is clear, thin, and entirely free of proteinaceous leakage or inflammatory exudate.
2. **Proximal Convoluted Tubules (PCT) (Middle Panel):** The PCTs exhibit thick, highly eosinophilic (bright pink) cellular linings. Crucially, the inner luminal surfaces retain their fuzzy, intact brush borders, and there is no evidence of epithelial cells detaching or sloughing into the center of the tubule.
3. **Distal Convoluted Tubules (DCT) (Right Panel):** The DCTs are structurally sound, featuring their characteristic wider, clearer lumens. The nuclei are neatly arranged along the basement membrane, and the tubule centers are entirely free of the solid proteinaceous casts that obstructed the Disease Control tissues.

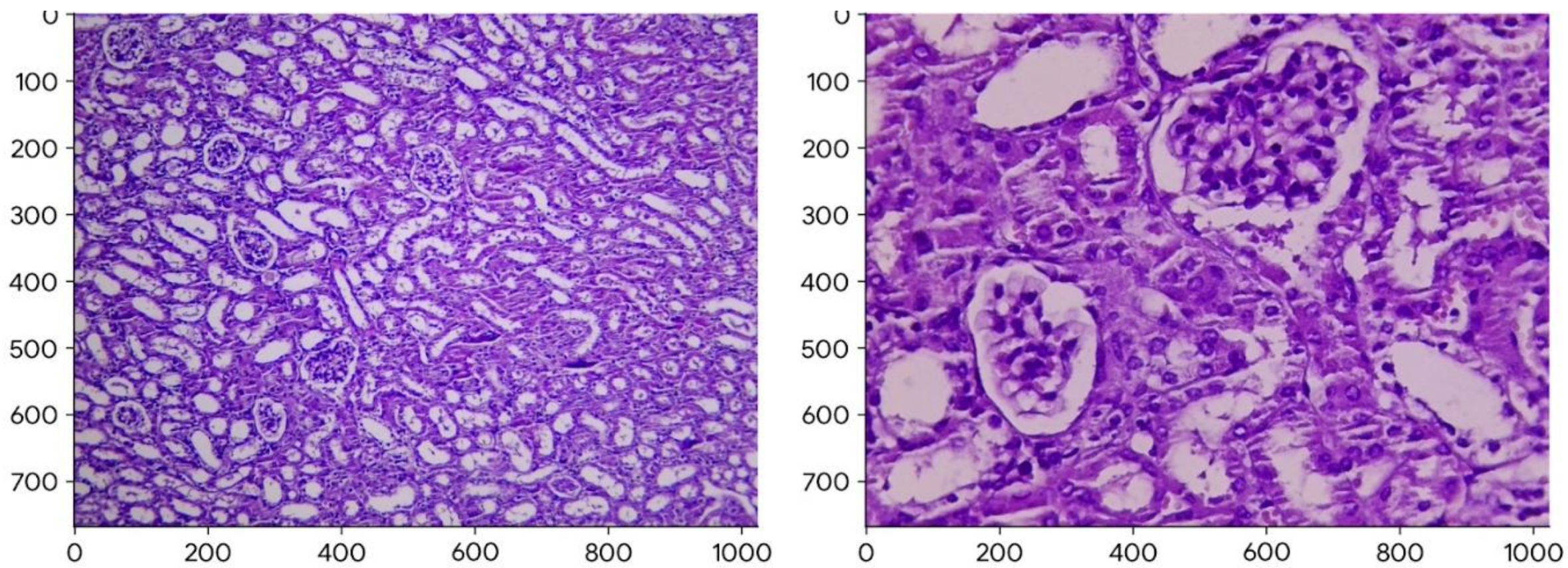

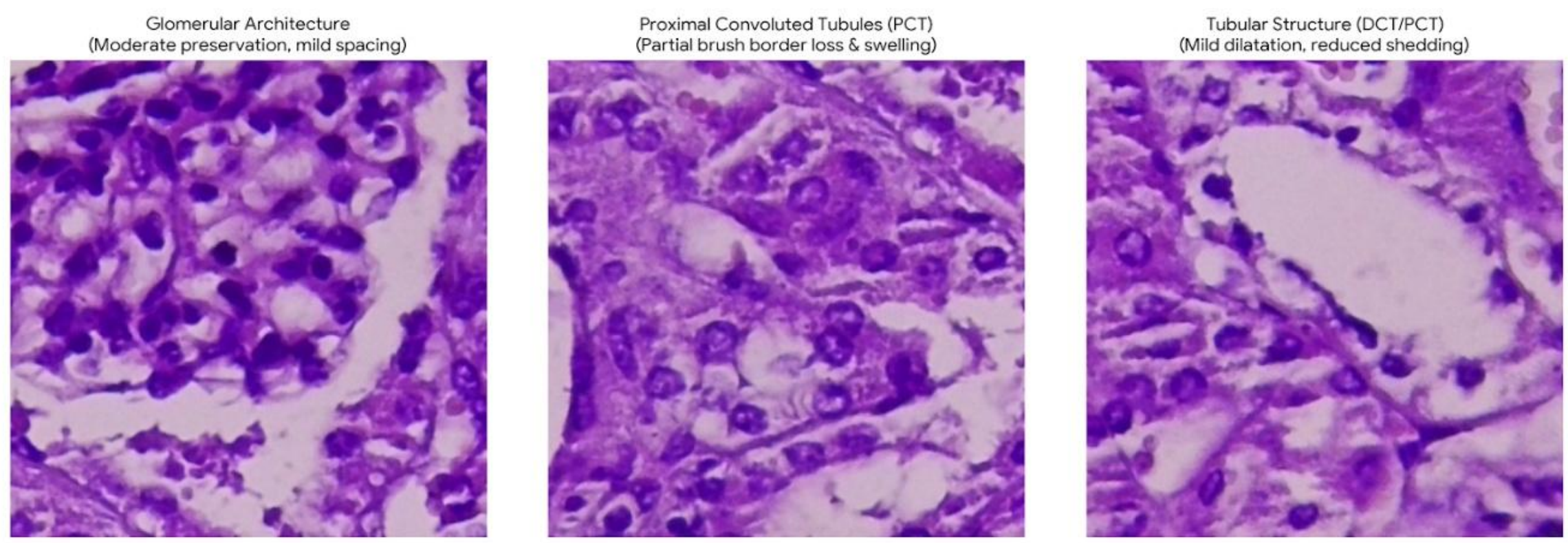

Supplementary Figure S4: Isolated Pathological Features of Prophylactic DMF Low-Dose (400x Magnification)

1. **Glomerular Architecture (Left Panel):** The glomerulus is moderately preserved. The capillary tuft has not entirely collapsed as it did in the Disease Control group, but Bowman's space shows mild widening and slight irregularities compared to the perfectly tight structure of the healthy controls.

2. **Proximal Convoluted Tubules (PCT) (Middle Panel):** This crop clearly illustrates the incomplete protection of the low dose. While the thick eosinophilic walls are mostly intact and severe desquamation is prevented, the cells exhibit mild swelling (cellular hypertrophy) and partial, patchy loss of the delicate apical brush borders.

3. **Tubular Structure (DCT/PCT) (Right Panel):** The distal and proximal tubules show signs of mild stress. Some lumens (like the prominent one in this crop) display mild to moderate dilatation. However, crucially, they remain largely free of the dense proteinaceous necrotic casts that completely plugged the nephrons in the untreated LPS subjects.

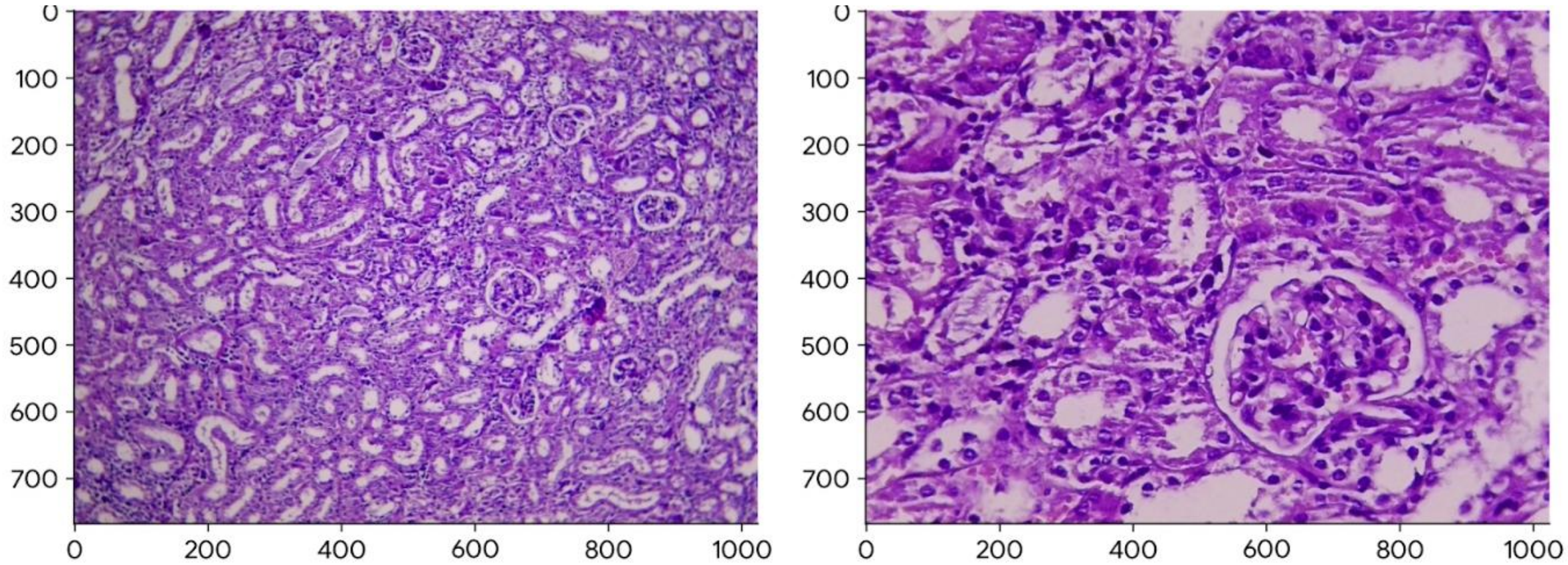

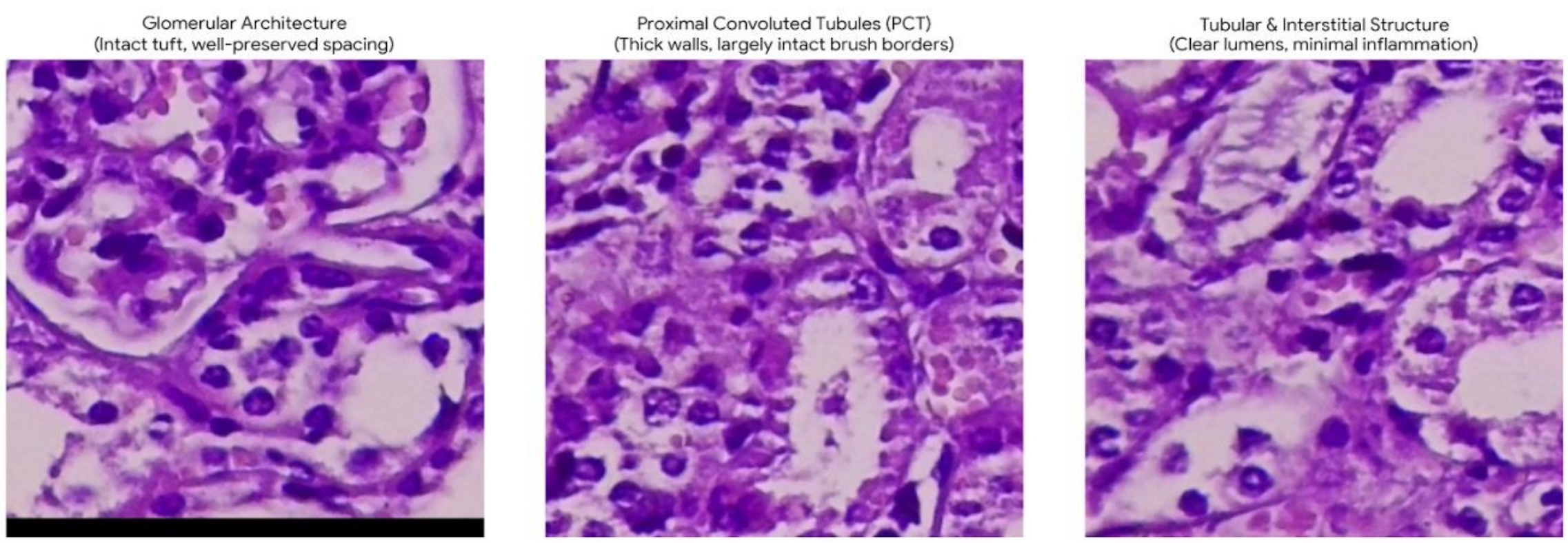

Supplementary Figure S5: Isolated Pathological Features of Prophylactic DMF High-Dose (400x Magnification)

1. **Glomerular Architecture (Left Panel):** The glomerulus is excellently preserved. The capillary tuft is full and intact, showing no signs of LPS-induced shrinkage. Bowman's space remains clear and is maintained at a normal physiological width, completely lacking the proteinaceous leakage seen in the diseased tissues.
2. **Proximal Convoluted Tubules (PCT) (Middle Panel):** This represents a significant improvement over the low-dose group. The PCTs feature thick, deeply eosinophilic cellular walls. Most importantly, the delicate inner brush borders are largely intact and well-preserved, and there is no evidence of the severe cellular sloughing or desquamation caused by LPS toxicity.
3. **Tubular & Interstitial Structure (Right Panel):** Both the proximal and distal tubules maintain their structural shape without significant swelling or dilatation. The lumens remain clear and unobstructed by necrotic debris or casts, proving that the high-dose DMF successfully prevented terminal cellular necrosis. Furthermore, the surrounding interstitial space is free of heavy inflammatory infiltration.

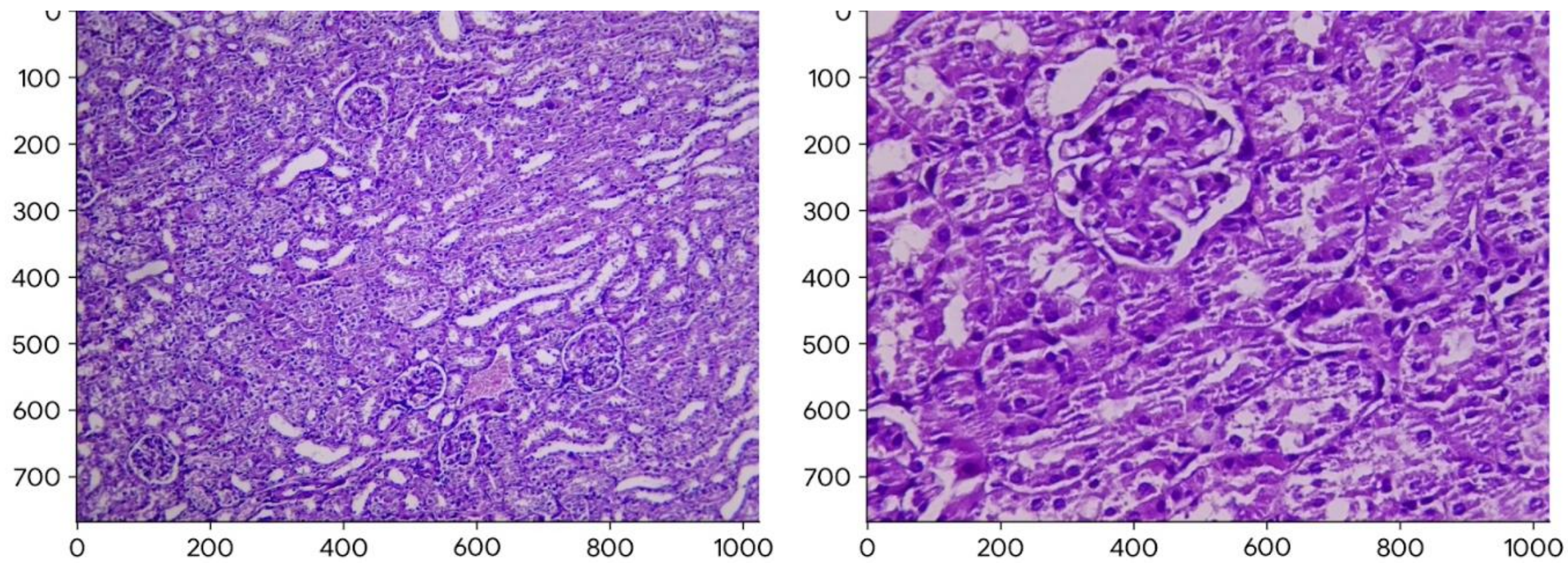

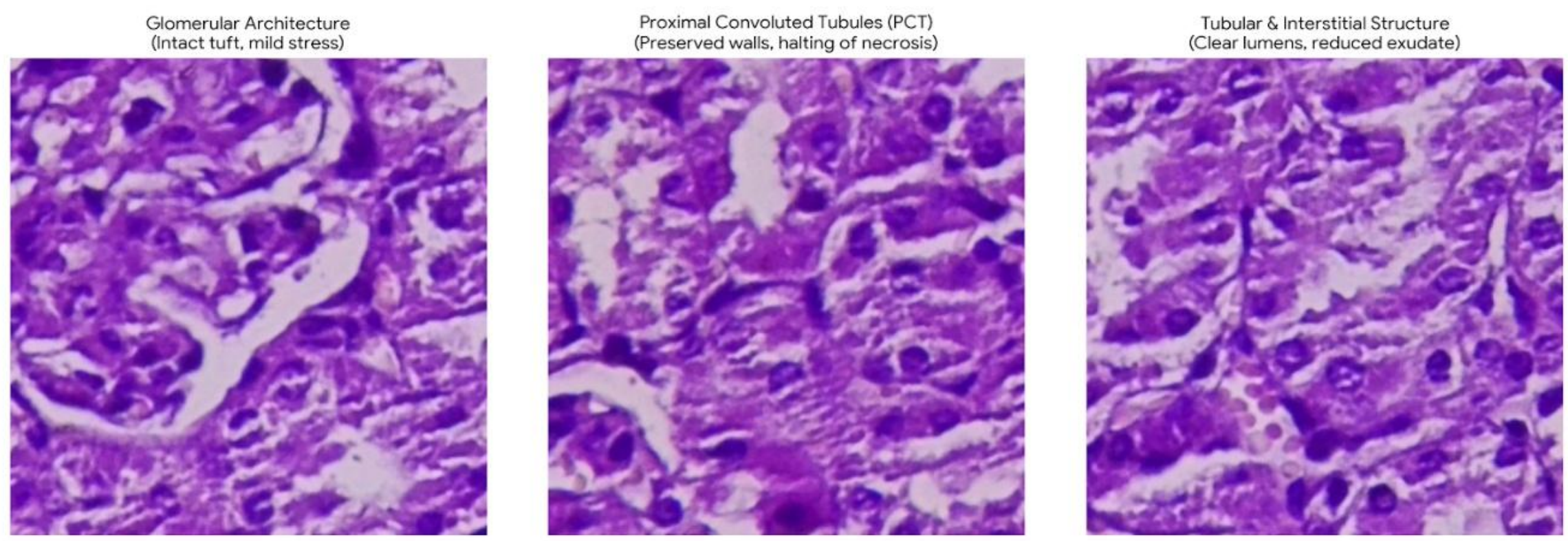

Supplementary Figure S6: Isolated Pathological Features of Therapeutic Control (400x Magnification)

1. **Glomerular Architecture (Left Panel):** The glomerulus is fundamentally intact, and the capillary tuft remains plump, successfully avoiding the severe shrinkage seen in the untreated LPS group. Bowman's space is clear, with no significant proteinaceous fluid leakage, although very mild initial spacing alterations are visible.
2. **Proximal Convoluted Tubules (PCT) (Middle Panel):** The intervention successfully halted the progression of tubular necrosis. The PCTs feature preserved, thick eosinophilic walls. Crucially, the massive epithelial desquamation (cellular sloughing) has been prevented. While some cells show very mild swelling (hypertrophy) indicative of the initial LPS shock, they remain firmly anchored to the basement membrane.
3. **Tubular & Interstitial Structure (Right Panel):** The lumens of both the proximal and distal tubules remain clear and functionally unobstructed. The single Dexamethasone dose successfully stopped the formation of the dense necrotic casts that plugged the nephrons in the Disease Control group, and the interstitial spaces show minimal to no edema.

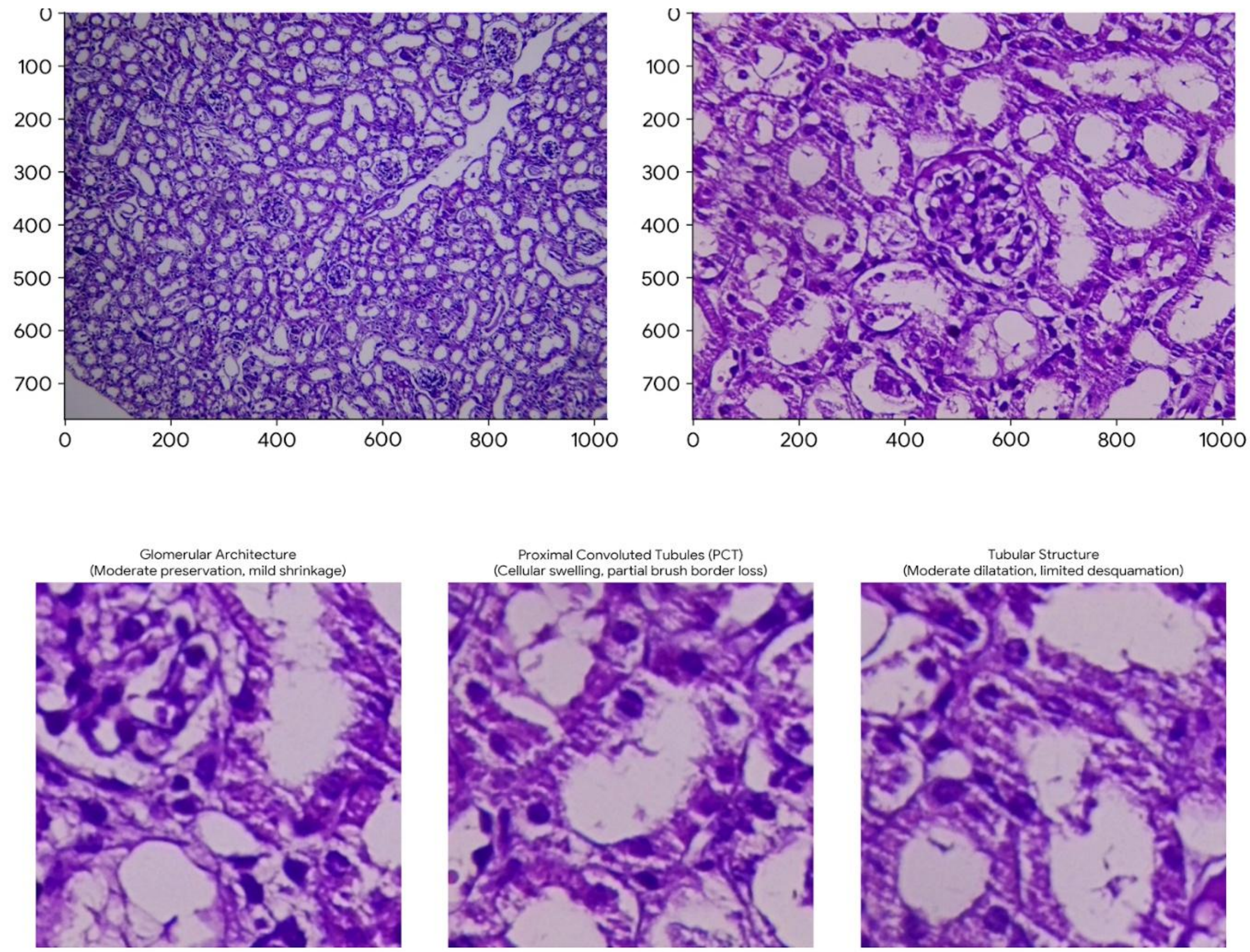

Supplementary Figure S7: Isolated Pathological Features of Therapeutic DMF Low-Dose (400x Magnification)

1. **Glomerular Architecture (Left Panel):** The glomerulus is moderately preserved but shows signs of early stress. The capillary tuft is slightly shrunken compared to the therapeutic control, and Bowman's space is mildly dilated.
2. **Proximal Convoluted Tubules (PCT) (Middle Panel):** The PCTs reflect a state of active injury mitigation. The massive epithelial desquamation (complete cellular sloughing) seen in the untreated group has been largely prevented. However, the epithelial cells are visibly swollen (hypertrophy) in response to the initial oxidative hit, and there is a noticeable, patchy loss of the protective apical brush borders along the luminal edges.
3. **Tubular Structure (Right Panel):** Several tubules exhibit moderate luminal dilatation. While the structural walls remain intact and attached to the basement membrane, the wide, empty spaces indicate significant osmotic stress. Crucially, however, the severe proteinaceous cast formations that signify terminal cell death in the Disease Control group are predominantly absent.

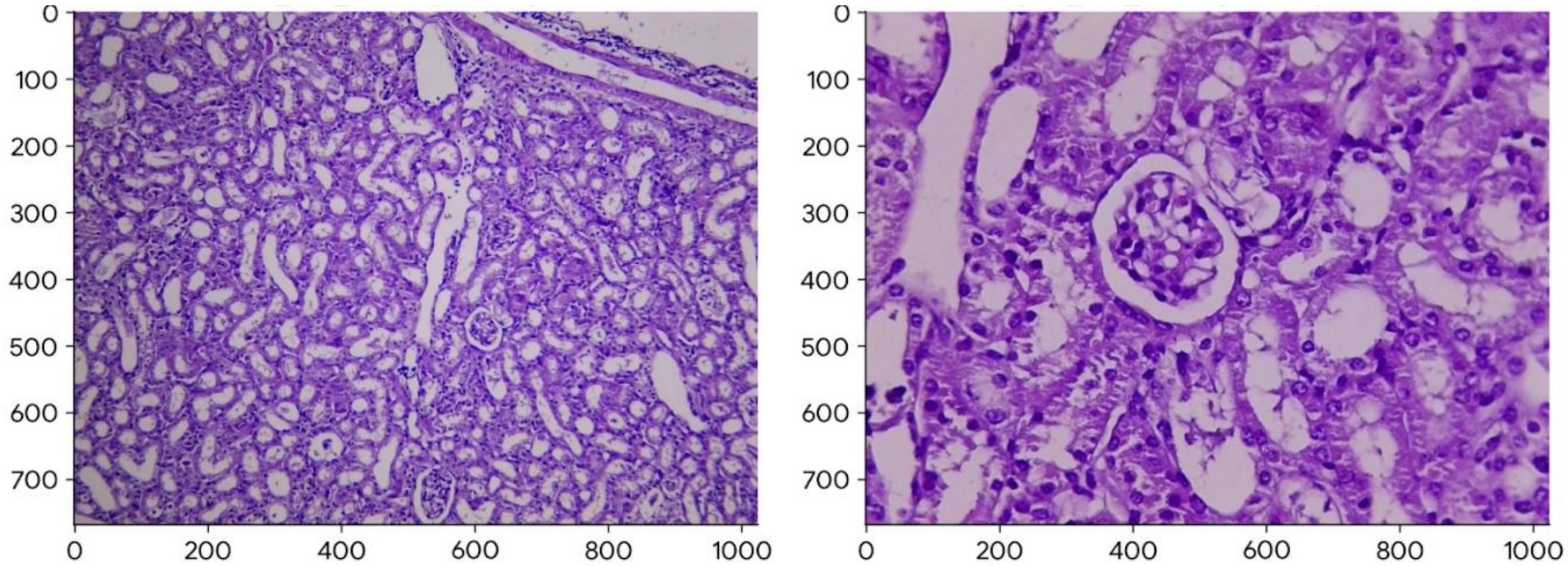

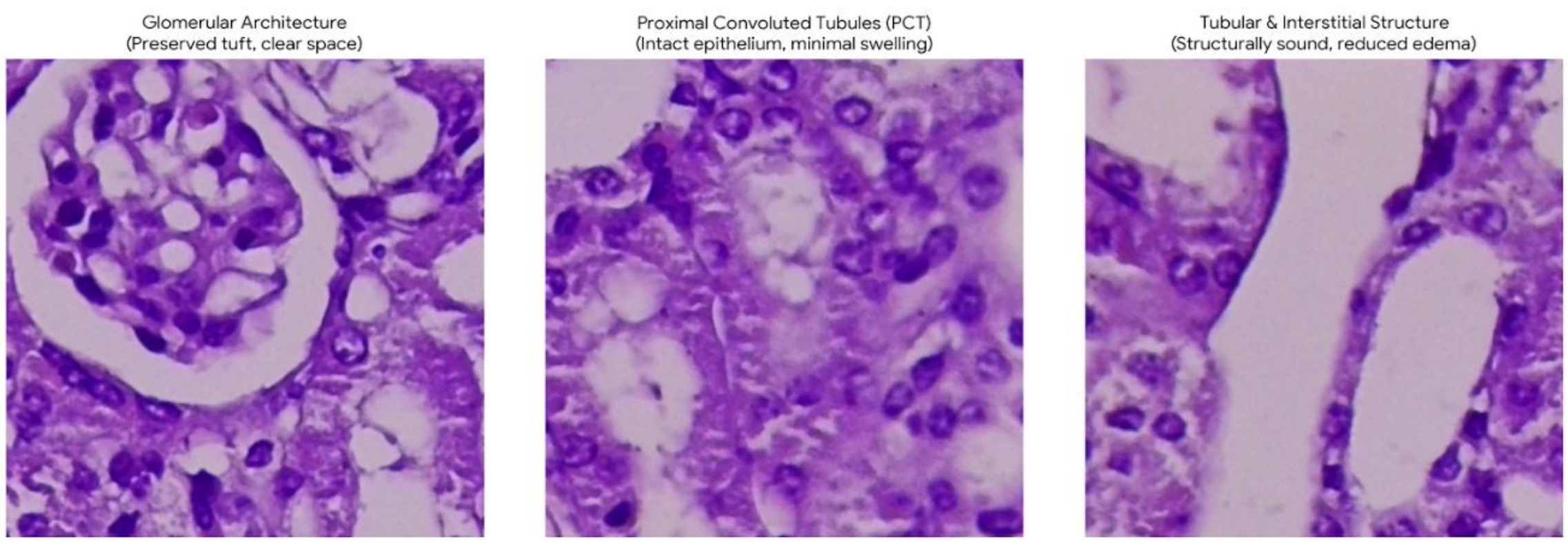

Supplementary Figure S8: Isolated Pathological Features of Therapeutic DMF High-Dose (400x Magnification)

At the cellular level, the ability of high-dose DMF to actively halt and reverse early necrotic signaling is clearly evident. I have isolated the key features for your comparative panel:

1. **Glomerular Architecture (Left Panel):** The glomerulus shows excellent structural integrity, indicating a strong rescue of the filtration barrier. The capillary tuft is robust and full, entirely lacking the shrinkage caused by LPS. Bowman's space is clear, distinct, and maintained at a physiological width without any fluid exudate
2. **Proximal Convoluted Tubules (PCT) (Middle Panel):** The high-dose intervention powerfully protected the vulnerable PCTs. The epithelial cells feature thick, healthy eosinophilic walls and maintain their tight attachment to the basement membrane, completely preventing terminal desquamation. Furthermore, the cellular swelling (hypertrophy) seen in the low-dose group is highly minimized, and the delicate apical brush borders are much better preserved.
3. **Tubular & Interstitial Structure (Right Panel):** The surrounding tubular networks are structurally secure. The lumens remain open, clear, and totally devoid of necrotic cellular debris and solid proteinaceous casts that plagued the untreated Disease Control. The surrounding interstitial matrix is tight, proving the drug actively shut down the microvascular leakage leading to edema.
